# Altered functional brain network connectivity and glutamate system function in transgenic mice expressing truncated *Disrupted-in-Schizophrenia 1*

**DOI:** 10.1038/tp.2015.60

**Published:** 2015-05-19

**Authors:** N Dawson, M Kurihara, D M Thomson, C L Winchester, A McVie, J R Hedde, A D Randall, S Shen, P A Seymour, Z A Hughes, J Dunlop, J T Brown, N J Brandon, B J Morris, J A Pratt

**Affiliations:** 1 Strathclyde Institute of Pharmacy and Biomedical Science, University of Strathclyde, Glasgow, UK; 2 Centre for Neuroscience (CeNsUS), University of Strathclyde, Glasgow, UK; 3 Psychiatric Research Institute for Neuroscience in Glasgow (PsyRING), Universities of Glasgow and Strathclyde, Glasgow, UK; 4 School of Physiology and Pharmacology, University of Bristol, Bristol, UK; 5 Institute of Neuroscience and Psychology, College of Medical, Veterinary and Life Sciences, University of Glasgow, Glasgow, UK; 6 Pfizer Neuroscience Research Unit, Cambridge, MA, USA; 7 University of Exeter Medical School, University of Exeter, Hatherly Laboratories, Exeter, UK; 8 School of Medical Sciences, Institute of Medical Sciences, University of Aberdeen, Aberdeen, UK; 9 Regenerative Medicine Institute, School of Medicine, National University of Ireland, Galway, Ireland

## Abstract

Considerable evidence implicates *DISC1* as a susceptibility gene for multiple psychiatric diseases. *DISC1* has been intensively studied at the molecular, cellular and behavioral level, but its role in regulating brain connectivity and brain network function remains unknown. Here, we utilize a set of complementary approaches to assess the functional brain network abnormalities present in mice expressing a truncated *Disc1* gene (*Disc1tr* Hemi mice). *Disc1tr* Hemi mice exhibited hypometabolism in the prefrontal cortex (PFC) and reticular thalamus along with a reorganization of functional brain network connectivity that included compromised hippocampal–PFC connectivity. Altered hippocampal–PFC connectivity in *Disc1tr* Hemi mice was confirmed by electrophysiological analysis, with *Disc1tr* Hemi mice showing a reduced probability of presynaptic neurotransmitter release in the monosynaptic glutamatergic hippocampal CA1–PFC projection. Glutamate system dysfunction in *Disc1tr* Hemi mice was further supported by the attenuated cerebral metabolic response to the NMDA receptor (NMDAR) antagonist ketamine and decreased hippocampal expression of NMDAR subunits 2A and 2B in these animals. These data show that the *Disc1* truncation in *Disc1tr* Hemi mice induces a range of translationally relevant endophenotypes underpinned by glutamate system dysfunction and altered brain connectivity.

## Introduction

Multiple independent linkage and association studies in diverse populations support a role for *Disrupted-in-Schizophrenia 1* (*DISC1*) as a genetic risk factor in a range of major mental illnesses, including schizophrenia, depression and bipolar disorder.^1^ Arguably, the most persuasive evidence supporting a role for *DISC1* in mental illness was gained from the initial studies of a large Scottish pedigree. These investigations showed that *DISC1* was disrupted by a balanced chromosomal translocation t(1;11)(q42;q14.3) that co-segregated with schizophrenia, depression and bipolar disorder.^2, 3, 4^ In this Scottish pedigree, the putative truncation of the *DISC1* protein is likely a key molecular event contributing to the increased risk of psychiatric disease, although other molecular alterations including the formation of aberrant fusion proteins between *DISC1* and *Boymaw/FP11*, a gene found on chromosome 11, could also be critical.^5, 6^ Intriguingly, all family members with the *DISC1* translocation, regardless of clinical diagnosis, display an impaired P300 event-related neurophysiological response, indicating that the genomic rearrangement leads to modified brain function.^4^ However, at present a significant gap remains in our understanding of how *DISC1* regulates brain function and connectivity.

*DISC1* has been implicated in a variety of neuronal processes including cell morphogenesis and migration during neural development,^7, 8, 9, 10, 11^ and the regulation of synaptic morphology.^12, 13, 14, 15^ A number of different mouse models have been developed and characterized to elucidate how the multiple functions of *DISC1* relate to aspects of brain development, function and various aspects of animal behavior. These models comprise spontaneous mutations, N-ethyl-N-nitrosurea generated point mutations, transgenic overexpression of mutant forms of *Disc1*, mice with targeted disruptions of specific exons in the *Disc1* gene and *in utero* knockdown of *Disc1* with RNA interference (summarized in Brandon and Sawa^1^ and Pratt *et al.*^16^). A range of behavioral phenotypes that is generally consistent with a role for *DISC1* in certain disease-relevant affective and cognitive processes has been reported in these mouse models.^16, 17, 18, 19, 20^ Previously, we developed a transgenic mouse model expressing a truncated form of *Disc1 (Disc1tr* hemizygous (Hemi) mice) and have shown that these animals exhibit a range of neural and behavioral phenotypes with translational relevance to schizophrenia and affective disorder.^18^ Here we use this mouse model to experimentally bridge the gap in our understanding regarding the role of *DISC1* in regulating system-level connectivity in the brain.

Emerging analytical methodologies now exist that allow for the close alignment of systems-level alterations in functional brain connectivity between both clinical and preclinical data sets. Recent brain imaging studies show altered functional brain network structure and regional functional connectivity in a range of psychiatric disorders including schizophrenia, major depression and bipolar disorder.^21, 22, 23, 24^ In addition, these analytical methods have recently been applied to elucidate how *DISC1* single-nucleotide variants impact on structural brain networks in humans.^25^ These techniques are now being applied to functional brain imaging data gained in preclinical models relevant to these psychiatric disorders.^26, 27, 28^ Hence, we sought to exploit these methods to characterize the impact of the *Disc1* truncation on functional brain network connectivity. Guided by these results, we utilized electrophysiological methods to probe the neurophysiology underlying one of these alterations in regional functional connectivity, reduced hippocampal–prefrontal cortex (PFC) connectivity, including characterization of the glutamatergic hippocampal–PFC projection.

In parallel with utilizing emerging technical and analytical approaches to gain a greater understanding of the circuit deficits in these mice, we also sought to understand the role of perturbed glutamatergic tone in this system, with a particular focus on *N*-methyl-d-aspartic acid receptor (NMDAR) function. Data from genetic,^29, 30, 31, 32, 33, 34^ epigenetic^35, 36^ and post-mortem expression studies^37, 38^ implicate a role for NMDAR dysfunction in the etiology of schizophrenia. In addition, the potential role of the NMDAR in schizophrenia is also supported by a wealth of pharmacological studies. Both acute and repeated exposure to NMDAR antagonists induces schizophrenia-like symptoms in humans^39, 40^ and acute administration of the NMDAR antagonist ketamine can exacerbate symptoms in schizophrenia patients.^41, 42^ Furthermore, a multitude of preclinical studies suggest that both acute and repeated NMDAR antagonist administration induces behavioral deficits with translational relevance to schizophrenia.^26, 43, 44, 45^ However, whether *Disc1* truncation impacts on NMDAR function *in vivo* is currently unknown. To address this gap in our knowledge, we characterized the impact of the NMDAR antagonist ketamine on regional cerebral metabolism in *Disc1tr* Hemi mice.

Through these studies we have been able to define deficits in brain function and functional connectivity in a genetic mouse model with relevance to a range of psychiatric disorders. In addition, we identify alterations in glutamate system function, including perturbed NMDAR expression, which may directly contribute to these alterations in brain function and functional connectivity. The congruence in the data sets gives us confidence that we have established a set of translationally relevant endophenotypes that could be used in the future for psychiatric drug discovery.

## Materials and methods

### Animals

*Disc1tr* transgenic mice were generated with a bacterial artificial chromosome expressing truncated mouse *Disc1* in a mixed genetic background of CBA/CaCrl and C57BL/6JCrl.^18^ Progenies, which were confirmed free of the *Nnt* and *Snca* mutations associated with the C57BL/6JCrl and C57BL/6JOlaHsd sub-strains, respectively, were backcrossed with mutation-free C57BL/6JRccHsd mice for nine generations, resulting in *Disc1tr* Hemi mice. C57BL/6JRccHsd mice were continuously used in all subsequent breeding to generate experimental *Disc1tr* Hemi mice and wild type (Wt) littermate controls. All experiments were completed in mice aged 12–20 weeks. Animals were singly housed under standard conditions (21 °C, 45–65% humidity, 12 h:12 h dark/light cycle (lights on 0600 hours) with food and water available *ad libitum*. All experiments were carried out in compliance with the UK Animals (Scientific Procedures) Act 1986.

### ^14^C-2-deoxyglucose (^14^C-2-DG) autoradiographic functional brain imaging

Group sizes for the ^14^C-2-deoxyglucose (^14^C-2-DG) autoradiographic brain imaging experiment were Wt *n*=20 (male *n*=10, female *n*=10) and *Disc1tr* Hemi *n*=20 (male *n*=10, female *n*=10). Local cerebral glucose utilization (LCGU) measurement was initiated 1 min after treatment with 30 mg  kg^-1^ (R,S)-ketamine (Sigma-Aldrich, Gillingham, Dorset, UK; in 2 ml kg^-1^ saline, intraperitoneally, *n*=20) or physiological saline (*n*=20) in accordance with previously published protocols^28, 46^ and detailed in the Supplementary Material.

### Analysis of functional brain network connectivity

Functional brain network structure and regional functional connectivity were analyzed only in control (saline treated) Wt and *Disc1tr* Hemi mice to avoid the potentially confounding influence of ketamine treatment on these measures.^28, 46^ The application of brain network analysis to ^14^C-2-DG brain imaging data has previously been described,^27, 28^ and the relevant levels of analysis are detailed further in the Supplementary Material. These algorithms allow us to quantitatively define the properties of brain networks at the global level (mean degree (*k*), average path length (Lp), clustering coefficient (*C*_p_)) and also to define the relative importance of each brain region in the context of the entire brain network (centrality analysis; degree (*K*_c_), betweenness (*B*_c_), eigenvector (*E*_c_)). Following the identification of *Disc1tr* Hemi-induced alterations in regional importance (centrality), we sought to characterize the alterations in regional functional connectivity that underlie these alterations. To achieve this, we employed the partial least squares regression (PLSR) algorithm to define significant differences in the functional connectivity of defined 'seed' brain regions of interest (ROIs) to all other ROIs analyzed. The application of PLSR to functional ^14^C-2-DG brain imaging data and its interpretation have previously been outlined^26, 46^ and are further detailed in the Supplementary Material.

### Electrophysiology

Electrophysiological recordings were made from modified coronal medial PFC (mPFC) slices^47^ prepared from *Disc1tr* Hemi and Wt male mice euthanized by cervical dislocation. Whole-cell voltage and current clamp recordings were made from visually identified neurons located in layer V or VI of the mPFC. Synaptic responses were elicited by stimulating a fiber bundle arising from the hippocampal formation.^47^ Full details are included in the Supplementary Material.

### NMDA receptor subunit expression

Expression levels of NMDAR subunits GluN1, GluN2A, GluN2B and GluN3B in the PFC and hippocampus were measured using standard western blot techniques. Full details are included in the Supplementary Material.

## Results

### Alterations in constitutive cerebral metabolism in *Disc1tr* Hemi mice

**Figure 1:**
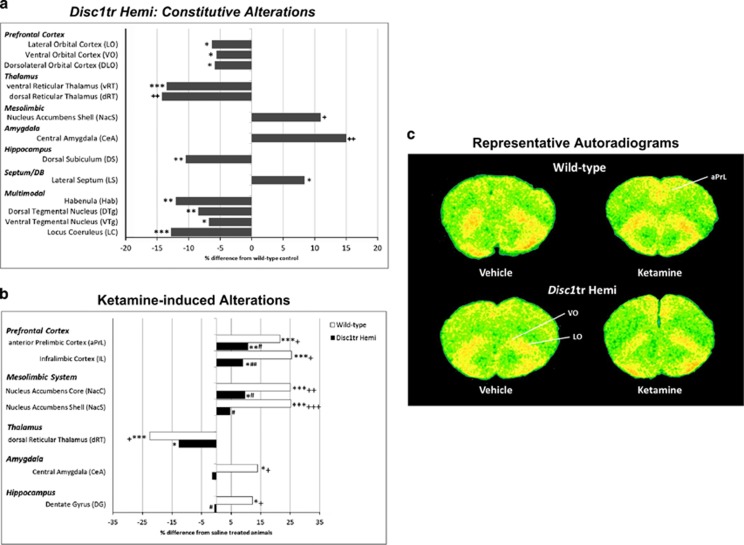
Altered constitutive cerebral metabolism and an attenuated metabolic response to ketamine treatment in *Disc1tr* Hemi mice. (**a**) Data shown as % difference in LCGU in *Disc1tr* Hemi mice relative to Wt littermates. *P<0.05, **P<0.01 and ***P<0.001, significant effect of genotype (main effect, two-way ANOVA (% difference between pooled saline and ketamine treatment groups)). ^+^*P*<0.05 and ^++^*P*<0.01, significant genotype effect in a brain region where a significant genotype × treatment effect was found by two-way ANOVA (pairwise *t*-test with Bonferroni–Holm correction, significant between saline-treated animals of the different genotypes (% difference between saline-treated animals shown)). (**b**) Ketamine-induced alterations in cerebral metabolism are attenuated in *Disc1tr* Hemi mice. ^+^*P*<0.05, ^++^*P*<0.01 and ^+++^*P*<0.001, significant genotype × treatment interaction as determined by two-way ANOVA. *P<0.05, **P<0.01 and ***P<0.001, a significant effect of ketamine within genotype (*t*-test with Bonferroni-Holm *post hoc* correction). ^#^*P*<0.05 and ^##^*P*<0.01, significant difference between ketamine-treated animals of the different genotypes (*t*-test with Bonferroni–Holm correction). (**c**) Representative pseudocolor autoradiograms from saline- and ketamine-treated *Disc1tr* Hemi animals and their Wt littermates. Warmer colors (red/yellow) denote increased levels of metabolism and colder colors (green/blue) denote lower levels of metabolism. Full data for constitutive and ketamine-induced alterations in LCGU are shown in the Supplementary Tables S2A–F. ANOVA, analysis of variance; LCGU, local cerebral glucose utilization; Wt, wild type.

Human functional neuroimaging studies suggest metabolic abnormalities in schizophrenia patients, including PFC hypometabolism (hypofrontality).^48, 49, 50, 51, 52^ Here we analyzed constitutive LCGU using ^14^C-2-DG autoradiography in *Disc1tr* Hemi and Wt mice. Using quantitative image analysis we identified significant alterations in 13 of the 58 ROIs measured in *Disc1tr* Hemi mice (Figure 1a; Supplementary Tables S1). *Disc1tr* Hemi mice exhibited functional hypofrontality, as indicated by significant hypometabolism in multiple orbital subfields of the PFC (dorsolateral orbital, ventral orbital and lateral orbital). In addition, significant hypometabolism was observed in the reticular thalamus (dorsal (dRT) and ventral reticular thalamus), habenula (Hab), hippocampal dorsal subiculum and in multiple neuromodulatory nuclei including the locus coeruleus and tegmental nuclei (dorsal and ventral). By contrast, constitutive LCGU was significantly increased in *Disc1tr* Hemi mice in the nucleus accumbens shell, central amygdala and lateral septum.

### Attenuated cerebral metabolic responses to ketamine in *Disc1tr* Hemi mice

As we have previously observed,^28, 46^ acute administration of ketamine leads to profound alterations in LCGU, most prominently PFC hypermetabolism. In *Disc1tr* Hemi mice, the cerebral metabolic response to ketamine treatment was significantly attenuated in 8 of the 58 ROIs analyzed (Figures 1b and c; Supplementary Table S2A and F). This included a significant attenuation in ketamine-induced PFC hypermetabolism (hyperfrontality; evident in the anterior prelimbic and infralimbic subfields) and of ketamine-induced hypermetabolism in the nucleus accumbens (core and nucleus accumbens shell). Although the LCGU response to ketamine was significantly attenuated in many ROIs in *Disc1tr* Hemi mice, in others the response was similar to that seen in Wt animals. This included ketamine-induced thalamic hypometabolism (evident in the anteroventral, mediodorsal, ventrolateral and ventral reticular thalamus) and hypometabolism in multiple neuromodulatory nuclei (median raphé, dorsal tegmental nuclei and ventral tegmental nuclei).

### Altered functional brain network structure in *Disc1tr* Hemi mice

**Figure 2:**
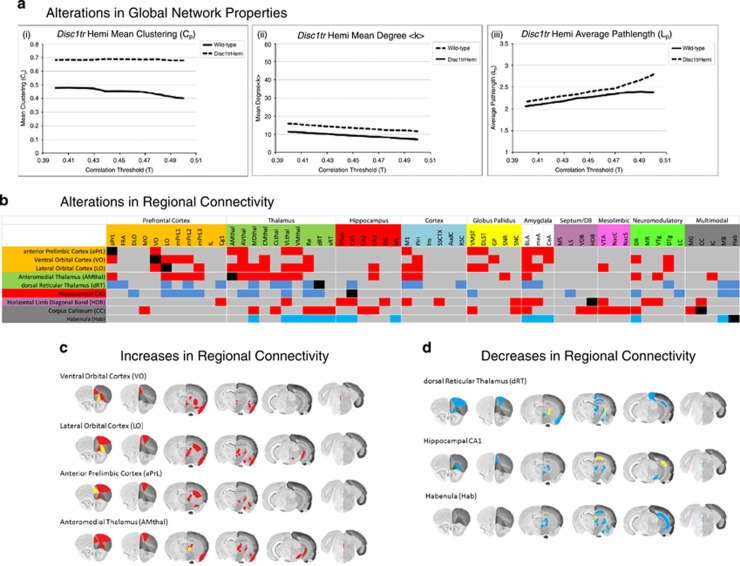
*Disc1tr* Hemi mutation-induced alterations in functional brain network properties and regional functional connectivity. (**a**) The mean clustering coefficient was significantly increased (*C*_p_; A(i), *P*=0.039) in the functional brain network of *Disc1tr* Hemi mutant mice. Mean degree (<*k*> A(ii)) and average path length (L_p_; A(iii)) are not significantly altered in the functional brain networks of *Disc1tr* Hemi mice. Significance was determined by comparison of the difference for each measure in the real networks relative to 55 000 random permutations of the real data across the entire correlation threshold range. (**b**) Heatmap showing how *Disc1tr* Hemi mutation alters the functional connectivity of 'seed' brain regions; in this case 'seed' regions (aPrL, LO, VO, AMthal, dRT, CA1, HDB and CC) were those found to have altered importance in the functional brain network as defined through centrality analysis. Red denotes a significant increase in functional connectivity of the seed region to a given region, whereas blue denotes a significant decrease in functional connectivity, as determined by statistical comparison of the variable importance to the projection statistic (*t*-test with Bonferroni *post hoc* correction) determined through PLSR analysis. Significance was set at *P*<0.05. Full data for each 'seed' region are shown in the Supplementary Tables S4–S12. (**c**) Brain images showing the anatomical localization of brain regions with significantly increased connectivity to selected prefrontal and thalamic 'seed' brain regions (aPrL, LO, VO and AMthal). Yellow denotes the anatomical localization of the 'seed' brain region and red denotes a significant increase in connectivity with the defined 'seed' region. (**d**) Brain sections showing the anatomical localization of brain regions with significantly decreased connectivity to selected 'seed' brain regions (dRT, CA1 and Hab). Yellow denotes the anatomical localization of the 'seed' brain region, blue denotes a significant decrease in connectivity with the defined 'seed' region. Brain section figures are modified from the Allen mouse brain atlas (mouse.brain-map.org/static/atlas). PLSR, partial least squares regression.

The constitutive LCGU data obtained in our studies provided us with the opportunity to characterize functional brain connectivity using a number of analytical approaches, including the use of algorithms from the emerging field of network science.^27, 28, 53^ As LCGU determined by ^14^C-2-DG autoradiography largely reflects the metabolic demands of synapses in the defined ROI,^54^ our connectivity measures reflect the functional relationship that exists in synaptic activity between brain regions. Applying these algorithms allowed us to quantitatively define the properties of functional brain networks at a number of scales, from the regional to global. On a global scale, clustering was significantly increased in the brain networks of *Disc1tr* Hemi mice, as evidenced by a significant increase in the mean clustering coefficient (*C*_p_, Figure 2a(i), *P*=0.039), suggesting an abnormal enhancement of functional connectivity between locally connected brain regions in *Disc1tr* Hemi mice. By contrast, the number of connections in the functional brain network of *Disc1tr* Hemi mice, as measured by mean degree ((*k*), Figure 2a(ii)), was unchanged, as was the efficiency of information transfer on a global scale, as shown by average path length (L_p_, Figure 2a(iii)). Overall, these data suggest that although the number of connections in the functional brain network of *Disc1tr* Hemi mice is not significantly altered, there is a pronounced reorganization of the functional brain network that results in an increased efficiency in local information transfer (also known as ‘cliquishness').

### Regional importance is significantly altered in the functional brain networks of *Disc1tr* Hemi mice

Centrality measures allow us to quantitatively elucidate the relative importance of each brain region in the context of the entire functional brain network and to determine how this is altered in *Disc1tr* Hemi mice. We found that the relative importance and functional connectivity of multiple PFC subfields (ventral orbital, lateral orbital and anterior prelimbic, Table 1) and their projecting thalamic nuclei (anteromedial thalamic nucleus) was significantly increased in *Disc1tr* Hemi mice. In addition, the horizontal limb of the diagonal band of Broca and corpus callosum also showed significantly increased importance in the brain networks of *Disc1tr* Hemi mice. By contrast, the importance and functional connectivity of the Hab, dRT and the CA1 subfield of the hippocampus were significantly decreased in the brain networks of *Disc1tr* Hemi mice as compared with Wt animals. These data suggest that the *Disc1tr* Hemi mutation has a profound influence on the relative importance of multiple brain regions within the context of brain networks. The increased importance and connectivity of PFC subfields and their projecting thalamic nuclei may contribute to the significantly increased local connectivity (increased clustering coefficient, Figure 2a(i)) seen in the brain networks of *Disc1tr* Hemi mice. Full data for centrality analysis are included in the Supplementary Material (Supplementary Table S3). To further elucidate how the functional connectivity of these regions is altered in *Disc1tr* Hemi mice, we employed PLSR analysis^26, 46^ to characterize how the connectivity of these ROIs to other neural subsystems is altered in the brain of *Disc1tr* Hemi mice.

### *Disc1tr* Hemi mutation-induced alterations in regional functional connectivity

Regional connectivity analysis, through the application of the PLSR algorithm,^26, 46^ identified significantly increased PFC–thalamic functional connectivity in the brains of *Disc1tr* Hemi mice (with the exception of the dRT, which showed decreased connectivity to the PFC). All three PFC subfields identified as showing significantly increased centrality in the brain networks of *Disc1tr* Hemi mice (anterior prelimbic, lateral orbital and ventral orbital) showed increased functional connectivity to multiple thalamic nuclei in these animals (Figures 2b and c). In addition, all three of these PFC subfields showed significantly increased functional connectivity to other PFC subfields and to the amygdala nuclei. Increased PFC–thalamic connectivity in *Disc1tr* Hemi mice was further supported when the anteromedial thalamic nucleus was considered as the 'seed' region, as this thalamic region showed significantly increased connectivity to multiple PFC subfields and to other thalamic nuclei (Figures 2b and d). These observations are consistent with the increased global clustering (Figure 2a(i)) seen in the brain networks of *Disc1tr* Hemi mice. In addition to these alterations, the functional connectivity of the horizontal limb of the diagonal band of Broca to multiple hippocampal subfields, and of the corpus callosum to multiple thalamic nuclei and components of the mesolimbic system, were significantly increased in *Disc1tr* Hemi mice (Figure 2b). By contrast, PLSR analysis of the three regions showing significantly decreased centrality in *Disc1tr* Hemi mice, the dRT, Hab and hippocampal CA1, showed reduced connectivity to multiple thalamic nuclei and to the PFC (Figures 2b and d). In particular, the dRT and Hab both showed a loss of connectivity to hippocampal regions, and both the hipppocampal CA1 and dRT showed reduced connectivity with multiple subfields of the PFC. In addition, all three of these regions showed a significant reduction in functional connectivity to each other in *Disc1tr* Hemi mice. Full data for the analysis of regional connectivity are shown in the Supplementary Material (Supplementary Tables S4–S12).

### Electrophysiological analysis confirms impaired hippocampal–PFC functional coupling in *Disc1tr* Hemi mice

**Figure 3:**
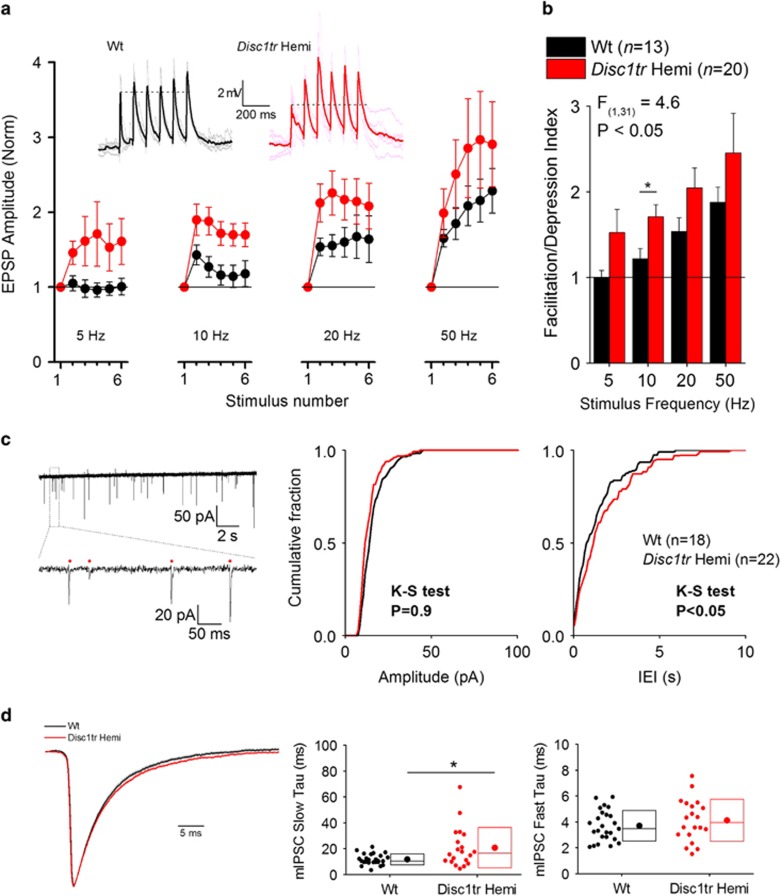
Altered intrinsic and network properties of medial prefrontal cortex (mPFC) neurons in *Disc1tr* Hemi mice. (**a**) Representative traces are EPSPs in response to a train of six stimuli delivered at 10 Hz. Faint traces are individual responses and bold traces are arithmetic means of the individual responses. The dotted line shows the amplitude of the first response for reference. Note the substantial facilitation in the *Disc1tr* Hemi neuron when compared with the Wt neuron. The graphs are pooled data from 13 Wt and 20 *Disc1tr* Hemi neurons, showing the EPSP facilitation/summation properties in response to trains of six stimuli delivered at various frequencies. EPSP amplitude is plotted as a fraction of the amplitude of the 1st EPSP in a train of six. (**b**) A facilitation/depression index was calculated as the normalized integral of the curves in **c**. These data show that *Disc1tr* Hemi neurons exhibit significantly greater levels of facilitation compared with Wt neurons, particularly in the theta frequency range (F_(1,93)_=4.6, *P*<0.05; repeated measures two-way ANOVA, *P<0.05 *post hoc* Bonferroni test). (**c**) The example trace showing sEPSCs recorded under voltage clamp from a Wt layer-V mPFC neuron. The expanded portion illustrates individual sEPSCs traces. The graphs are cumulative probability histograms showing the average distribution of sEPSC amplitudes (left) and IEIs (right). The IEI probability distribution of *Disc1tr* Hemi sEPSCs is significantly right shifted (*P*<0.05, K–S test), suggesting a reduction in release probability, whereas the amplitude of sEPSCs was similar between genotypes. (**d**) Mean normalized to peak miniature inhibitory postsynaptic current (mIPSC) traces from Wt and *Disc1tr* Hemi neurons. Shaded area denotes s.e.m. Decay curves were fitted with a double-exponential curve to calculate slow and fast tau. These data illustrate that *Disc1tr* Hemi mIPSCs have a significantly slower slow tau as compared with their Wt counterparts (*P<0.05; Mann–Whitney *U*-test). ANOVA, analysis of variance; EPSP, excitatory postsynaptic potential; IEI, inter-event interval; K–S test, Kolmogorov–Smirnov test; mPFC, medial prefrontal cortex; sEPSC, spontaneous excitatory postsynaptic current; Wt, wild type.

Our regional functional connectivity analysis showed that connectivity between a select number of brain regions is impaired in *Disc1tr* Hemi mice, including reduced hippocampal CA1–mPFC connectivity (Figures 2b and d). The mPFC receives direct glutamatergic input from the CA1 subfield of the hippocampus.^55^ Therefore, given the altered CA1–mPFC connectivity and the attenuated response to the NMDAR antagonist ketamine present in *Disc1tr* Hemi mice, we sought to explore the contribution of this glutamatergic projection to this functional connectivity deficit using a recently developed modified coronal brain slice preparation, which preserves hippocampal synaptic connections onto mPFC neurons (see Parent *et al.*^47^ and Supplementary Information). In this preparation, trains of electrical stimulation (six pulses at 5, 10, 20 or 50 Hz) applied to hippocampal afferent fibers produce glutamatergic excitatory postsynaptic potentials in layer V/VI cells in the mPFC. As the stimulus train progresses, the peak depolarization elicited by each excitatory postsynaptic potential increases with stimulus number. This increase in synaptically driven depolarization arises both from short-term synaptic plasticity (that is, frequency facilitation) and summation of temporally adjacent responses. Interestingly, we found greater levels of excitatory postsynaptic potential facilitation when stimulus trains were applied to slices from *Disc1tr* Hemi mice, particularly at 10 Hz (in the rodent theta frequency range; Figure 3a). To quantify the level of frequency facilitation, we derived a facilitation/depression index from the normalized amplitude curves by calculating the normalized integral of each curve recorded from every cell. Thus, a facilitation/depression index of 1 represents no net change in synaptic response during the six-pulse train.

These data, summarized in Figure 3b, illustrate that on average *Disc1tr* Hemi hippocampal–mPFC synapses express significantly greater levels of short-term frequency facilitation than Wt synapses (two-way repeated measures analysis of variance: main effect of genotype F_(1,31)_=4.6, *P*=0.04), suggesting a reduction in release probability at the hippocampal–mPFC glutamatergic synapse.^56^ A *post hoc* pairwise comparison indicated that frequency facilitation was significantly enhanced in *Disc1*tr Hemi neurons in response to trains delivered at 10 Hz (*P*=0.02) but not at other frequencies.

Additional whole-cell patch clamp recordings from layer V/VI pyramidal neurons in the mPFC were employed to further examine deficits in mPFC function. We initially looked at cell-intrinsic excitability properties. Neurons were subdivided into two broad categories, regular spiking and intrinsic bursting, based on the respective absence or presence of a fast after-depolarizing potential. The passive membrane properties (resting membrane potential, input resistance, membrane time constant and sag potential) of both regular spiking and intrinsic bursting cell types were not altered in *Disc1tr* Hemi mice. Furthermore, the neuronal excitability properties (the number of spikes elicited by 500-ms depolarizing current injections of various amplitudes) and the properties of the first action potential recorded in response to a 300-pA current injection for both cell types were not altered in *Disc1tr* Hemi mice (Supplementary Information; Supplementary Table S13; Supplementary Figure S1). Local network connectivity has previously been reported to be altered in other models of altered *DISC1* function.^57, 58, 59^ To examine the effect of truncated *Disc1* in *Disc1tr* Hemi mice, we recorded spontaneous excitatory postsynaptic currents (sEPSCs) under voltage clamp conditions from Wt and *Disc1tr* Hemi pyramidal neurons. Although the probability distribution of the amplitude of sEPSCs was similar in Wt and *Disc1tr* Hemi neurons (*P*=0.34, Kolmogorov–Smirnov test), the distribution of the inter-event intervals of the sEPSCs was significantly decreased in *Disc1tr* Hemi mice (Figures 3c, *P*<0.05, Kolmogorov–Smirnov test). Thus, the mean of the median sEPSC inter-event intervals in Wt neurons was 0.8±0.1 s, whereas in *Disc1tr* Hemi neurons it was 1.2±0.3 s. These data suggest that synaptic release probability is reduced in *Disc1tr* Hemi mPFC neurons. However, when we examined miniature EPSCs and IPSCs (in the presence of 500 nM TTX and internal Cs ions), no significant difference in miniature EPSC and IPSC frequency or amplitude was observed (Supplementary Information; Supplementary Figure S2). This suggests that although there is an alteration in network function, the functional status of the fundamental synaptic release machinery is largely unaffected. On closer inspection of the miniature IPSC kinetics, neurons in *Disc1tr* Hemi mice have a slower component in decay kinetics (slow tau) in comparison with those from Wt animals (Figure 3d).

### Western blot analysis indicates altered hippocampal NMDA receptor subunit expression in *Disc1tr* Hemi mice

**Figure 4:**
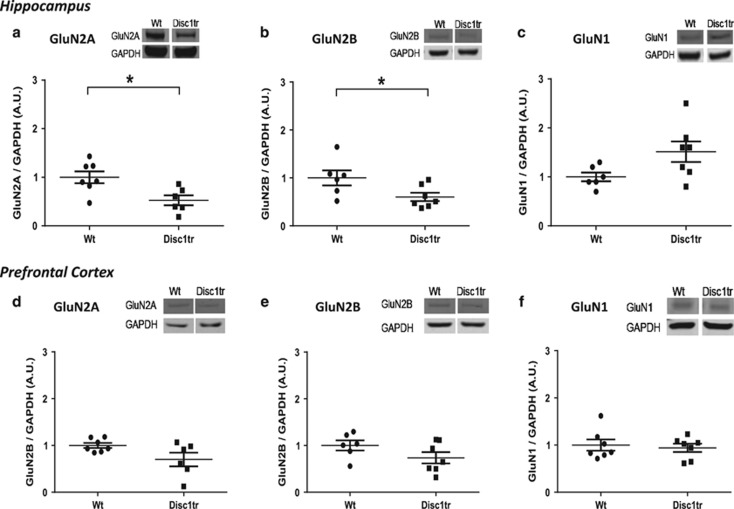
Altered NMDA receptor (NMDAR) subunit protein expression in the hippocampus of *Disc1*tr Hemi mice. Figures show expression levels of NMDAR subunits GluN2A, GluN2B and GluN1 in hippocampus (**a**–**c**) and prefrontal cortex (PFC, **d**–**f**) of *Disc1tr* Hemi mice (*n*=7) relative to wild type (Wt, *n*=7) littermates. Data were normalized to glyceraldehyde 3-phosphate dehydrogenase (GAPDH) and are shown as mean±s.e.m. *P<0.05, significantly different from Wt (*t*-test). In the hippocampus, levels of GluN2A (*P*=0.01) and GluN2B (*P*=0.04) were significantly lower in *Disc1tr* Hemi mice compared with Wt littermates. There was a non-significant trend for GluN1 to be higher in the hippocampus of *Disc1tr* Hemi mice (*P*=0.057). In the PFC there were no significant differences between the two genotypes (*P*>0.05).

Analysis of NMDAR subunit expression in Wt and *Disc1tr* Hemi mice revealed significant effects of genotype on GluN2A and GluN2B expression in the hippocampus but not in the PFC (Figure 4). Levels of hippocampal GluN2A (*P*=0.01) and GluN2B (*P*=0.04) protein expression were both significantly decreased in *Disc1tr* Hemi mice (Figures 4a and b), whereas there was a trend (*P*=0.057) toward increased GluN1 levels in the hippocampus of *Disc1*tr Hemi mice (Figure 4c). In contrast, GluN1, GluN2A and GluN2B protein expression levels were not significantly altered in the PFC of *Disc1tr* Hemi mice (Figures 4d and f). In addition, we found that GluN3B protein expression levels were not significantly altered in either the hippocampus or PFC of *Disc1*tr Hemi mice (data not shown). The findings for GluN1 and GluN2B were both replicated with two different antibodies (data not shown).

## Discussion

Our data show that *Disc1* truncation induces translational endophenotypes in mice that are relevant to those seen in psychiatric diseases. This includes schizophrenia-related alterations in brain function, functional brain network connectivity and glutamate system function. Our findings encompass both hypofrontality and a compromised hippocampal–PFC functional connectivity, observations that parallel alterations seen in the brains of schizophrenia patients. In addition, *Disc1*tr Hemi mice show glutamatergic dysfunction and an NMDAR hypoactivation consistent with the glutamatergic hypofunction hypothesis of schizophrenia. Arguably, the molecular insult present in these animals more closely resembles that present in the human *DISC1* pedigree^18^ than those present in some of the other *Disc1* mutant mouse models currently available. Although this preclinical model does not recapitulate the full complexity of the genetic alterations present in these individuals^5, 6^ our data strongly suggest that disruption of *Disc1* is a key molecular event contributing to the emergence of disease-relevant alterations in brain function. The relevance of the *Disc1tr* Hemi model to other forms of *DISC1* mutations associated with psychiatric disease, such as missense mutations in the gene, should also be interpreted with some caution. Other *Disc1* mutant mouse models that are currently available may be more relevant to the effects of these specific mutations.

### Effect of *Disc1* truncation on brain function and functional connectivity

We have, for the first time, shown that *Disc1* truncation modifies constitutive brain function, inducing hypofrontality (PFC hypometabolism in orbitofrontal regions), and hypometabolism in the RT and hippocampus. Recently, orbitofrontal cortex pathology has been shown in mice expressing a putative dominant-negative form of C-terminal truncated *Disc1* (DN-*Disc1* mice),^60^ suggesting that this brain region may be particularly susceptible to *Disc1* dysfunction. In functional brain imaging studies, patients with schizophrenia exhibit reduced metabolic activity, relative to control subjects, in the PFC (hypofrontality), including orbitofrontal cortex hypofunction,^49^ along with hypometabolism in temporal cortical areas, and in the mediodorsal and anteroventral thalamic nuclei.^51, 52^ Metabolic abnormalities in thalamic nuclei and the temporal lobe have been shown to correlate with the severity of positive symptoms, whereas dysfunction in the PFC has been correlated with severity of the negative symptoms and cognitive deficits of schizophrenia.^48, 50, 51, 61^ Thus the metabolic imaging endophenotype of *Disc1tr* Hemi mice suggests the existence of an altered pattern of brain activity that, in humans, would be linked to the full range of symptom domains of schizophrenia. Although there is no direct evidence for RT hypofunction in psychiatric patients, perhaps because the region is beyond the resolution of imaging techniques in humans, emerging evidence does support RT dysfunction in schizophrenia.^62^ In addition, this brain region is not only directly implicated in the regulation of attentional processing^63^ and sensorimotor gating,^64^ processes known to be disrupted in psychiatric disease, but the region also largely consists of GABAergic parvalbumin-positive neurons, a primary cell type that has been shown to be dysfunctional in many brain regions, including the thalamus, in schizophrenia patients.^65, 66, 67, 68^ Furthermore, in rodents *Disc1* expression is high in this region^69^ and we have previously identified this region as being hypofunctional in preclinical models relevant to psychiatric disorders based on prolonged NMDAR hypofunction (subchronic phencyclidine (PCP) treatment),^26, 70, 71^ in which RT parvalbumin expression levels are also decreased.^70, 72^ RT hypoactivity in *Disc1tr* Hemi mice therefore raises the possibility that compromised activity of this region contributes to the susceptibility of developing psychiatric disease in situations where *DISC1* function is impaired. *Disc1* has been shown to localize to mitochondria^73, 74^ and has an important role in regulating various aspects of mitochondrial function, including mitochondrial trafficking and subcellular localization, mitochondrial calcium dynamics and adenosine triphosphate production.^5, 75, 76, 77^ The potential contribution of mitochondrial dysfunction to the reduced cerebral metabolism seen in *Disc1*tr Hemi mice certainly warrants further systematic investigation. Elucidating the regulatory mechanisms that link *Disc1tr*-induced alterations in mitochondrial dysfunction and the brain region-specific alterations in cerebral metabolism, and functional connectivity, seen in *Disc1tr* Hemi mice, would be of particular interest.

For the first time we have shown that the expression of truncated *Disc1* significantly modifies the organization of functional brain networks, resulting in the reduced functional connectivity and influence of key brain regions (dRT, Hab and hippocampal CA1), including alterations that support reduced hippocampal–PFC functional connectivity. This observation is consistent with recent human data showing that a nonsynonymous single-nucleotide polymorphism in *DISC1*, which leads to a serine-to-cysteine substitution at amino-acid 704 in the *DISC1* protein and is associated with schizophrenia, alters hippocampal–PFC functional connectivity during memory encoding.^78^ Furthermore, functional integration between these neural subsystems is disrupted in schizophrenia patients.^79^ In addition, our electrophysiology data support reduced efficacy of the hippocampal–PFC glutamatergic projection as a driver of the reduced functional connectivity seen between these neural systems in *Disc1tr* Hemi mice. This could also result from the developmental effects of the mutant *Disc1*, leading to impaired corticogenesis and/or neuronal migration.^8, 9, 10, 11, 18^ Alternatively, errors in axonal targeting, which have been reported in another *Disc1* mutant mouse line expressing truncated *Disc1* (*Disc1*^*tm1kara*^ mice),^80^ would also be predicted to have a detrimental effect on regional connectivity in key pathways, such as the monosynaptic glutamatergic hippocampal–mPFC projection. Decreased connectivity in this projection is not only consistent with the emerging central role of this projection in psychiatric disease^81^ but also with the decreased synchrony seen between these neural systems in other genetic (22q11; D*f*(16)*A*^+/^^−^ mice)^82^ and neurodevelopmental (methylazomethanol acetate (MAM-E17)-treated rats)^83^ rodent models relevant to these diseases.

Another key finding of our study was the widespread evidence for abnormally increased functional connectivity between the PFC and thalamus as a result of *Disc1* truncation. Schizophrenia is characteristically linked to reduced PFC–thalamic connectivity^84^ and we have recently reported that acute NMDAR blockade leads to decreased PFC–thalamus functional connectivity in mice.^46^ However, there are also reports supporting enhanced PFC–thalamic connectivity in schizophrenia.^85, 86^ Furthermore, enhanced thalamocortical connectivity is not only seen in major depression^87^ but also in autism,^88^ a developmental disorder for which *DISC1* has also been identified as a genetic risk factor.^89, 90^ Hence, the *Disc1* truncation present in *Disc1tr* Hemi mice may induce aspects of altered brain connectivity relevant to multiple psychiatric disorders, an effect congruent with the increased risk of developing both schizophrenia and affective disorders in humans with the translocation in *DISC1*.^2, 3, 4^

### Effect of the *Disc1tr* mutation on neuronal electrophysiological properties and the relationship to brain connectivity data

Our functional connectivity analysis, derived from LCGU studies, suggested that functional connectivity between various brain regions in *Disc1tr* Hemi mice is disrupted. As the signal detected by the ^14^C-2-DG brain imaging protocol used in our study largely reflects the metabolic demands of synapses within a given ROI,^54^ the functional connectivity measures derived from these data likely reflect synaptic functional connectivity between brain regions. Thus our functional connectivity data support disrupted synaptic connectivity between brain regions in *Disc1tr* Hemi mice. This suggestion is confirmed by the *in vitro* electrophysiological approaches used in our study, showing that excitatory glutamatergic inputs within the mPFC of *Disc1tr* Hemi mice are dysfunctional. Specifically, our data suggest that action potential-dependent spontaneous synaptic transmission is disrupted in the mPFC of *Disc1tr* Hemi mice. Importantly, this does not result from differences in quantal synaptic release probability, as miniature EPSC frequency and amplitude were unaffected in *Disc1tr* Hemi mice. This dichotomy could result from a functional uncoupling of presynaptic action potentials and neurotransmitter release mechanisms, consistent with recent evidence showing that mutant forms of *DISC1* alter presynaptic function and glutamate release.^58, 91^ These findings mesh well with our constitutive LCGU data, which are indicative of reduced metabolic activity in the PFC. Although our data support a key role for presynaptic dysfunction in the disrupted hippocampal–PFC connectivity seen in *Disc1tr* Hemi mice, it is also important to remember that alterations in brain structure, such as the decreased width of the corpus callosum that we have previously identified in these animals,^18^ may contribute to some of the deficits in functional connectivity seen in these animals.

Functional connectivity between temporal and frontal regions is disturbed in patients suffering from schizophrenia, which in turn contributes to deficiencies in working memory in these patients.^92, 93^ Furthermore, in rodents performing working memory tasks, local field potential theta oscillations in the hippocampus and mPFC become transiently synchronous,^94^ a phenomenon that is disrupted in other genetic mouse models of relevance to schizophrenia such as the 22q11 mouse model (specifically the D*f*(16)*A*^+/^^−^ line).^82^ We have used an *in vitro* slice preparation that preserves the hippocampal–mPFC synaptic pathway^47^ to study connectivity between these regions in *Disc1tr* Hemi mice. By delivering short trains of electrical stimuli to the hippocampal input, we were able to study the short-term synaptic dynamics of hippocampal–mPFC synapses. Interestingly, we found a significant enhancement of short-term synaptic plasticity that was particularly striking in the theta frequency range (10 Hz). These data are highly suggestive of a lower probability of Ca^2+^-dependent release at this specific synapse, but not more generally in the mPFC, further supporting a deficit in hippocampal–mPFC connectivity in *Disc1tr* Hemi mice. This mechanism, along with the altered balance in local mPFC inhibitory and excitatory neurotransmission that results from *Disc1* truncation,^57^ may directly contribute to the reduced hippocampal–mPFC functional connectivity seen in *Disc1*tr Hemi mice (Figure 2d). In this regard, we have also observed subtle changes in the kinetics of inhibitory synaptic transmission in *Disc1tr* Hemi mice (Figure 3d). Although the mechanisms underlying these changes are yet to be elucidated, our data, coupled with evidence of reduced parvalbumin staining in the PFC of these animals,^18^ suggest that local inhibitory circuits are also disrupted.

### Effect of *Disc1* truncation on glutamate system function

The attenuated functional metabolic response to ketamine observed in the *Disc1tr* Hemi mice suggests that NMDAR function is perturbed in these animals. Indeed, our western blot data are indicative of NMDAR hypofunction at the molecular level, for NMDARs containing the GluN2A and GluN2B subunits, in the hippocampus of *Disc1tr* Hemi mice (Figures 4a and b). Interestingly, similar effects on NMDAR subunit expression were recently reported in the hippocampus of mice with astrocyte-restricted expression of dominant-negative mutant *DISC1*,^95^ implying some specific functional relationship between *Disc1* and expression of these subunits. Furthermore, our electrophysiological data, showing reduced frequency facilitation in the direct glutamatergic hippocampal–mPFC projection in *Disc1*tr Hemi mice (Figure 3b), suggest that the probability of glutamate release from synapses is significantly reduced in *Disc1tr* Hemi mice, and this may also contribute to the reduced response to ketamine seen in these animals. The alterations in hippocampal NMDAR subunit expression seen in *Disc1*tr Hemi mice may directly contribute to the alterations in brain function and regional functional connectivity seen in these animals. Reduced GluN2A and GluN2B subunit expression in the hippocampus of *Disc1*tr Hemi mice may uncouple the regulation of hippocampal activity from that of its thalamic glutamatergic afferents, including that of the nucleus reuniens which act as a relay to the hippocampus from the PFC.^96^ Indeed, decreased thalamo–hippocampal functional coupling in *Disc1*tr Hemi mice is supported by our regional functional connectivity data (Figures 2b and d). This reduced thalamo–hippocampal connectivity could, in turn, directly contribute to the decreased hippocampal–PFC connectivity (Figures 2b and d) and reduced glutamate release from the direct hippocampal–mPFC projection, as supported by our electrophysiology data (Figure 3), seen in *Disc1* Hemi mice. Thus, the alterations in hippocampal NMDAR subunit expression seen in *Disc1tr* Hemi mice could, through the alterations in regional functional connectivity seen in these animals, induce a glutamatergic hypofunction in the PFC in the absence of alterations in PFC NMDAR subunit expression levels (Figures 4d and f). In addition, the altered connectivity in this thalamo–hippocampal–prefrontal circuit in *Disc1tr* Hemi mice could also contribute to the reduced ability of ketamine to induce PFC hypermetabolism in *Disc1tr* Hemi mice (Figure 1). Other alterations in functional connectivity also seen in *Disc1tr* Hemi mice could also contribute to this effect. For example, in our previous work we identified the RT as a primary locus for driving ketamine-induced hyperfrontality.^46^ Hence, the reduced RT–PFC functional connectivity present in *Disc1tr* Hemi mice may contribute to the attenuated impact of ketamine treatment on PFC metabolism in these animals.

The pattern of altered regional metabolism, including orbital cortex and RT hypometabolism, seen in *Disc1tr* Hemi mice closely resembles that in animals treated subchronically with the NMDAR antagonist PCP.^26^ Moreover, subchronic PCP treatment not only decreases hippocampal CA1–mPFC functional connectivity^26^ but also decreases the importance of the hippocampal CA1 region in functional brain networks.^27^ This parallels observations we have made in the brain networks of *Disc1tr* Hemi mice, as does the reduced importance of the RT and the compromised dRT–PFC functional connectivity that is seen in both *Disc1tr* Hemi mice and animals treated subchronically with PCP. The prolonged NMDAR hypofunction induced by subchronic PCP treatment also causes the functional segregation of the hippocampus–PFC in brain networks,^27^ consistent with the potential of NMDAR hypofunction to compromise integration between these neural systems in *Disc1tr* Hemi mice and with the decreased probability of synaptic release that we have identified in these animals in the hippocampal–PFC glutamatergic projection. The contribution of NMDAR and glutamate system dysfunction to the altered functional connectivity seen in *Disc1tr* Hemi mice is also consistent with data supporting a key role for deficits in NMDAR-mediated synaptic plasticity in the functional dysconnectivity seen in schizophrenia.^97, 98^ However, although many of the systems-level alterations seen in both the subchronic PCP model and *Disc1tr* Hemi mice are closely aligned, there are also some divergent observations that must also be considered. For example, subchronic PCP treatment does not induce the increase clustering, centered around increased thalamic and PFC connectivity, seen in *Disc1tr* Hemi mice.^27^ This suggests that other, non-NMDAR dependent, mechanisms are also important in mediating the impact of *Disc1* truncation on brain functional connectivity. This could include, for example, deficits in GABAergic neurotransmission, supported by our observation that miniature IPSC kinetics are altered in *Disc1*tr Hemi mice (Figure 3d), and this certainly warrants further systematic investigation.

Overall, these data provide valuable new insight into the systems-level alterations in functional brain connectivity that result from *Disc1* truncation and the mechanisms that underlie these alterations, including reduced presynaptic glutamate release and altered NMDAR function. The translational nature of the systems-level paradigms used here also offer new opportunities for future psychiatric drug discovery.

## Figures and Tables

**Table 1:** *Disc1tr* Hemi mutation-induced alterations in regional centrality

*Region*	*Centrality measure*	*Wild type*	*Disc1tr* Hemi
*Prefrontal cortex (PFC)*			
Ventral orbital (VO)	Degree	−1.04	2.43*
Lateral orbital (LO)	Degree	−1.05	3.96*
Anterior prelimbic (aPrL)	Degree	**−3.14**	0.54*
			
*Thalamus*			
Anteromedial thalamus (AMthal)	Degree	**−2.31**	2.40**
Dorsal reticular (dRT)	Degree	1.78	−3.44*
	Eigenvector	1.58	−3.93*
			
*Hippocampus*			
CA1	Eigenvector	1.68	−3.51*
			
*Septum/diagonal band of Broca*			
Horizontal limb DB (HDB)	Betweenness	−1.22	4.58*
			
*Multimodal*			
Habenula (Hab)	Degree	1.78	−3.28*
	Eigenvector	1.52	−3.97*
Corpus callosum (CC)	Betweenness	−1.32	4.48*

Data shown as the *z*-score value for the given centrality measure in the real network as compared with that in 11 000 calibrated random Erdös–Rényi networks. Bold denotes those regions defined as important hubs (*z*>1.96) or exteriorities (*z*<−1.96) in the relevant network. The significance of *Disc1tr* Hemi-induced alterations in regional importance was determined by comparing the real z-score difference between the groups relative to that of 11 000 random permutations of the raw data.

*P<0.05 and **P<0.01 denotes significant difference in centrality relative to wild type control. Full data for each centrality measure are shown in the Supplementary Table S3.

## Notes

At the time of the project JRH, PAS, ADR, ZAH, JD and NJB were employees of Pfizer. SS, BJM and JAP were recipients of funding as part of the Translational Medicine Research Collaboration. The remaining authors declare no conflict of interest.
