# Supplementary material for: Altered functional brain network connectivity and glutamate system function in transgenic mice expressing truncated Disrupted-in-Schizophrenia 1
Source: Transl Psychiatry. 2015 May 19;5(5):e569–. doi: 10.1038/tp.2015.60 (PMC4471291; doi:10.1038/tp.2015.60)
Supplement: Supplementary Information [file tp201560x1.doc]

**Dawson *et al.,* 2015. Supplementary Information**

Table S1. Blood glucose and tracer parameters in the 2-deoxyglucose imaging experiment

|  | **Wild-type** | | ***Disc1tr* Hemi** | |
| --- | --- | --- | --- | --- |
|  | **Saline** | **Ketamine** | **Saline** | **Ketamine** |
| Plasma ^14^C concentration (nCi/ml) | 23.98 ± 1.36 | 27.85 ± 2.05 | 24.69 ± 3.69 | 26.84 ± 1.26 |
| Plasma Glucose concentration (mmol/L) | 9.31 ± 0.43 | 10.43* ± 0.39 | 9.12 ± 0.18 | 10.29* ± 0.53 |
| Whole Brain Average ^14^C concentration (WBAv [nCi/mg]) | 127.59 ± 6.53 | 131.92 ± 11.10 | 157.74 ± 22.68 | 123.86 ± 9.65 |
| WBAv : Plasma ^14^C ratio | 5.39 ± 0.35 | 4.75 ± 0.31 | 7.43 ± 2.15 | 4.65 ± 0.34 |

*Data shown as mean ± S.E.M. While ketamine treatment significantly increases the plasma glucose concentration this does not significantly impact on the ability of the tracer to enter the brain, as supported by no significant alterations in the WBAv:^14^C Plasma ratio. In addition, genotype does not significantly affect blood glucose and tracer levels, the WBAv ^14^C concentration or the WBAv: Plasma ^14^C ratio. *denotes p<0.05 significant difference from saline-treated animals (main treatment effect, 2-way ANOVA).*

Table S2 (A). Abbreviations for brain regions of interest (RoI) analysed in the 2-deoxyglucose imaging study

| ***Prefrontal Cortex*** |  | ***Mesolimbic*** |  | ***Hippocampus*** |
| --- | --- | --- | --- | --- |
| Anterior Prelimbic Cortex (aPrL) |  | Ventral Tegmental Area (VTA) |  | Dorsal Subiculum (DSub) |
| Frontal Association Area (FRA) |  | Nucleus Accumbens Core (NacC) |  | Cornu Ammonis 1 (CA1) |
| Dorsolateral Orbital Cortex (DLO) |  | Nucleus Accumbens Shell (NacS) |  | Cornu Ammonis 2 (CA2) |
| Medial Orbital Cortex (MO) |  | ***Basal Ganglia*** |  | Cornu Ammonis 3 (CA3) |
| Lateral Orbital Cortex (LO) |  | Ventromedial Striatum (VMST) |  | Dentate Gyrus (DG) |
| Medial Prelimbic Cortex (Layer 1, [mPrL1]) |  | Dorsolateral Striatum (DLST) |  | Molecular Layer (ML) |
| Medial Prelimbic Cortex (Layer 2, [mPrL2]) |  | Globus Pallidus (GP) |  | ***Multimodal*** |
| Medial Prelimbic Cortex (Layer 3, [mPrL3]) |  | Substantia Nigra pars Reticulata (SNR) |  | Habenula (Hab) |
| ***Cortex*** |  | Substantia Nigra pars Compacta (SNC) |  | Mamillary Body (MB) |
| Cingulate Cortex (Cg1) |  | ***Amygdala*** |  | Corpus Callosum (CC) |
| Primary Motor Cortex (M1) |  | Basolateral Amygdala (BLA) |  | **Septum/Diagonal Band of Broca (DB)** |
| Piriform Cortex (Piri) |  | Central Amygdala (CeA) |  | Lateral Septum (LS) |
| Insular Cortex (Ins) |  | Medial Amygdala (MeA) |  | Medal Septum (MS) |
| Somatosensory Cortex (SSCTX) |  | ***Auditory*** |  | Horizontal Limb of DB (HDB) |
| Retrosplenial Cortex (RSC) |  | Inferior Colliculus (IC) |  | Vertical Limb of DB (VDB) |
| ***Thalamus*** |  | Medial Geniculate (MG) |  |  |
| Anteromedial Thalamus (AMthal) |  | Auditory Cortex (AudC) |  |  |
| Anteroventral Thalamus (AVthal) |  | ***Neuromodulatory*** |  |  |
| Mediodorsal Thalamus (MDthal) |  | Dorsal Raphe (DR) |  |  |
| Centromedial Thalamus (CMthal) |  | Median Raphe (MR) |  |  |
| Centrolateral Thalamus (CLthal) |  | Ventral Tegmental Nucleus (VTg) |  |  |
| Ventrolateral Thalamus (VLthal) |  | Dorsal Tegmental Nucleus (DTg) |  |  |
| Ventromedial Thalamus (VMthal) |  | Locus Coeruleus (LC) |  |  |
| Nucleus Reuniens (Re) |  |  |  |  |
| Dorsal Reticular Thalamus (dRT) |  |  |  |  |
| Ventral Reticualr Thalamus (vRT) |  |  |  |  |

Table S2(B) Overt alterations in local cerebral glucose utilization in saline and ketamine treated *Disc1tr* Hemi mice: prefrontal cortex and other cortical regions

| **Region** | **Wild-type (Wt)** | | | | | | ***Disc1tr* Hemi** | | | | | | **% Genotype Effect (Treatments pooled)** | **% Ketamine Effect (Genotypes pooled)** | | **% Genotype**  **Effect** | | **% Ketamine Effect** | |
| --- | --- | --- | --- | --- | --- | --- | --- | --- | --- | --- | --- | --- | --- | --- | --- | --- | --- | --- | --- |
|  | **Saline** | | | **Ketamine** | | | **Saline** | | | **Ketamine** | | |  |  |  |  |  |  |  |
|  | **Mean** | ± | **SEM** | **Mean** | ± | **SEM** | **Mean** | ± | **SEM** | **Mean** | ± | **SEM** |  |  |  | **Saline** | **Ketamine** | **Wt** | ***Disc1tr***  **Hemi** |
| *Prefrontal Cortex* | | | | | | | | | | | | | | | | | | | |
| **aPrL** | 1.20 | ± | 0.03 | **1.45^ΦΦΦ^** | ± | 0.03 | 1.18 | ± | 0.03 | **1.30^Δ^** | **±** | **0.02** | -6 | 16 | -1 | | -10 | 22 | **11^#^** |
| **FRA** | 1.23 | ± | 0.03 | 1.26 | ± | 0.03 | 1.21 | ± | 0.03 | 1.20 | ± | 0.03 | -3 | 1 | -1 | | -5 | 3 | -1 |
| **DLO** | 0.97 | ± | 0.03 | 1.04 | ± | 0.04 | 0.96 | ± | 0.02 | 0.94 | ± | 0.02 | **-6^+^** | 3 | -1 | | -10 | 7 | -2 |
| **MO** | 1.07 | ± | 0.03 | 1.24 | ± | 0.04 | 1.07 | ± | 0.03 | 1.11 | ± | 0.03 | -6 | **10***** | 0 | | -10 | 16 | 4 |
| **VO** | 1.56 | ± | 0.02 | 1.75 | ± | 0.05 | 1.50 | ± | 0.05 | 1.63 | ± | 0.03 | **-6^+^** | **11***** | -4 | | -7 | 12 | 9 |
| **LO** | 1.74 | ± | 0.02 | 2.00 | ± | 0.05 | 1.65 | ± | 0.07 | 1.85 | ± | 0.04 | **-6^+^** | **13***** | -5 | | -7 | 15 | 12 |
| **mPrL1** | 1.20 | ± | 0.04 | 1.56 | ± | 0.04 | 1.23 | ± | 0.03 | 1.52 | ± | 0.04 | 0 | **27***** | 2 | | -3 | 30 | 24 |
| **mPrL2** | 1.25 | ± | 0.03 | 1.50 | ± | 0.02 | 1.25 | ± | 0.03 | 1.40 | ± | 0.02 | -3 | **16***** | 0 | | -6 | 20 | 12 |
| **mPrL3** | 1.15 | ± | 0.03 | 1.40 | ± | 0.03 | 1.15 | ± | 0.02 | 1.34 | ± | 0.03 | -2 | **19***** | 0 | | -4 | 22 | 17 |
| **IL** | 0.94 | ± | 0.02 | **1.18^ΦΦΦ^** | ± | 0.03 | 0.98 | ± | 0.02 | **1.07^ΦΔΔ^** | ± | 0.02 | -3 | 17 | 4 | | -9 | 25 | **9^##^** |
| *Cortex* | | | | | | | | | | | | | | | | | | | |
| **Cg1** | 1.34 | ± | 0.04 | 1.42 | ± | 0.03 | 1.31 | ± | 0.03 | 1.34 | ± | 0.03 | -4 | 4 | -3 | | -6 | 6 | 3 |
| **M1** | 1.31 | ± | 0.03 | 1.17 | ± | 0.02 | 1.29 | ± | 0.03 | 1.19 | ± | 0.02 | 0 | **-9***** | -1 | | 2 | -10 | -8 |
| **Piri** | 1.57 | ± | 0.02 | 1.74 | ± | 0.06 | 1.56 | ± | 0.05 | 1.67 | ± | 0.04 | -3 | **9**** | -1 | | -4 | 11 | 7 |
| **Ins** | 1.09 | ± | 0.03 | 1.14 | ± | 0.02 | 1.07 | ± | 0.03 | 1.09 | ± | 0.04 | -3 | 3 | -2 | | -4 | 4 | 2 |
| **SSCTX** | 1.33 | ± | 0.02 | 1.05 | ± | 0.02 | 1.29 | ± | 0.03 | 1.08 | ± | 0.03 | -1 | **-18***** | -3 | | 3 | -21 | -16 |
| **RSC** | 1.59 | ± | 0.03 | 1.43 | ± | 0.04 | 1.50 | ± | 0.05 | 1.40 | ± | 0.03 | -4 | **-8**** | -6 | | -2 | -10 | -6 |

*Data shown as the Mean ± S.E.M ^14^C-2-deoxyglucose uptake ratio and relevant % changes. * denotes p<0.05, ** denotes p<0.01 and *** denotes p<0.001 significant treatment effect (2-way ANOVA, no genotype x treatment interaction). ^+^ denotes p<0.05 and ^++^ denotes p<0.01 significant genotype effect (2-way ANOVA, no genotype x treatment interaction). ^#^ denotes p<0.05 and ^##^ denotes significant genotype x treatment interaction (2-way ANOVA). ^Φ^ denotes p<0.05, ^ΦΦ^ denotes p<0.01 and ^ΦΦΦ^ denotes p<0.001 significant difference from saline control (within genotype, t-test with post-hoc Bonferroni-Holm correction). ^Δ^ denotes p<0.05, ^ΔΔ^ denotes p<0.01 and ^ΔΔΔ^ denotes p<0.001 significant genotype effect (within treatment group, t-test with Bonferroni-Holm correction). Post-hoc results are shown only for regions in which a significant genotype x treatment interaction was found by 2-way ANOVA.*

Table S2(C) Overt alterations in local cerebral glucose utilization in saline and ketamine treated *Disc1tr* Hemi mice: thalamic nuclei

| **Region** | **Wild-type (Wt)** | | | | | | **Disc1tr Hemi** | | | | | | **% Genotype Effect (Treatments pooled)** | **% Ketamine Effect (Genotypes pooled)** | **% Genotype Effect** | | **% Ketamine Effect** | |
| --- | --- | --- | --- | --- | --- | --- | --- | --- | --- | --- | --- | --- | --- | --- | --- | --- | --- | --- |
|  | **Saline** | | | **Ketamine** | | | **Saline** | | | **Ketamine** | | |  |  |  |  |  |  |
|  | **Mean** |  | **SEM** | **Mean** |  | **SEM** | **Mean** |  | **SEM** | **Mean** |  | **SEM** |  |  | **Saline** | **Ketamine** | **Wt** | **Disc1tr Hemi** |
| *Thalamus* | | | | | | | | | | | | | | | | | | |
| **AMthal** | 1.71 | ± | 0.05 | 1.55 | ± | 0.04 | 1.57 | ± | 0.06 | 1.55 | ± | 0.03 | -4 | -5 | -8 | 0 | -9 | -1 |
| **AVthal** | 1.75 | ± | 0.05 | 1.59 | ± | 0.04 | 1.63 | ± | 0.06 | 1.57 | ± | 0.03 | -4 | **-7*** | -7 | -1 | -9 | -4 |
| **MDthal** | 1.46 | ± | 0.04 | 1.36 | ± | 0.02 | 1.42 | ± | 0.06 | 1.35 | ± | 0.01 | -2 | **-6*** | -3 | -1 | -7 | -5 |
| **CMthal** | 1.11 | ± | 0.04 | 1.02 | ± | 0.04 | 1.10 | ± | 0.03 | 1.01 | ± | 0.03 | -1 | **-8*** | 0 | -1 | -8 | -8 |
| **CLthal** | 1.22 | ± | 0.02 | 1.20 | ± | 0.03 | 1.21 | ± | 0.03 | 1.27 | ± | 0.02 | 2 | 1 | -1 | 6 | -2 | 5 |
| **VLthal** | 1.52 | ± | 0.04 | 1.23 | ± | 0.03 | 1.50 | ± | 0.05 | 1.26 | ± | 0.02 | 0 | **-17***** | -1 | 3 | -19 | -16 |
| **VMthal** | 1.43 | ± | 0.03 | 1.26 | ± | 0.03 | 1.40 | ± | 0.05 | 1.29 | ± | 0.02 | 0 | **-10***** | -3 | 3 | -12 | -8 |
| **Re** | 1.19 | ± | 0.08 | 1.08 | ± | 0.05 | 1.29 | ± | 0.08 | 1.17 | ± | 0.06 | 8 | -9 | 8 | 8 | -9 | -9 |
| **dRT** | 1.19 | ± | 0.04 | **0.92^ΦΦΦ^** | ± | 0.02 | **1.02^ΔΔ^** | ± | 0.05 | **0.89^Φ^** | ± | 0.02 | -9 | -18 | -14 | -3 | -22 | **-13^#^** |
| **vRT** | 1.20 | ± | 0.04 | 0.98 | ± | 0.04 | 1.01 | ± | 0.04 | 0.88 | ± | 0.03 | **-13^+++^** | **-16***** | -16 | -10 | -18 | -13 |

*Data shown as the Mean ± S.E.M.^14^C-2-deoxyglucose uptake ratio and relevant % changes. * denotes p<0.05, ** denotes p<0.01 and *** denotes p<0.001 significant treatment effect (2-way ANOVA, no genotype x treatment interaction). ^+^ denotes p<0.05 and ^+ +^denotes p<0.01 significant genotype effect (2-way ANOVA, no genotype x treatment interaction). ^#^denotes p<0.05 significant genotype x treatment interaction (2-way ANOVA). ^Φ^ denotes p<0.05, ^ΦΦ^ denotes p<0.01 and ^ΦΦΦ^ denotes p<0.001 significant difference from saline control (within genotype, t-test with post-hoc Bonferroni-Holm correction). ^Δ^ denotes p<0.05, ^ΔΔ^ denotes p<0.01 and ^ΔΔΔ^ denotes p<0.001 significant genotype effect (within treatment group, t-test with Bonferroni-Holm correction). Post-hoc results are shown only for regions in which a significant genotype x treatment interaction was found by 2-way ANOVA.*

Table S2(D) Overt alterations in local cerebral glucose utilization in saline and ketamine treated *Disc1tr* Hemi mice: mesolimbic, basal ganglia and amygdala regions

| **Region** | **Wild-type (Wt)** | | | | | | ***Disc1tr* Hemi** | | | | | | **% Genotype Effect (Treatments pooled)** | **% Ketamine Effect (Genotypes pooled)** | **% Genotype Effect** | | **% Ketamine Effect** | |
| --- | --- | --- | --- | --- | --- | --- | --- | --- | --- | --- | --- | --- | --- | --- | --- | --- | --- | --- |
|  | **Saline** | | | **Ketamine** | | | **Saline** | | | **Ketamine** | | |  |  |  |  |  |  |
|  | **Mean** |  | **SEM** | **Mean** |  | **SEM** | **Mean** |  | **SEM** | **Mean** |  | **SEM** |  |  | **Saline** | **Ketamine** | **Wt** | ***Disc1tr* Hemi** |
| *Mesolimbic* | | | | | | | | | | | | | | | | | | |
| **VTA** | 1.24 | ± | 0.02 | 1.13 | ± | 0.02 | 1.19 | ± | 0.04 | 1.10 | ± | 0.03 | -3 | **-9***** | -4 | -2 | -9 | -8 |
| **NacC** | 0.88 | ± | 0.01 | **1.10^ΦΦΦ^** | ± | 0.03 | 0.93 | ± | 0.02 | **1.02^ΦΔ^** | ± | 0.03 | -2 | 17 | 6 | -7 | 25 | **10^##^** |
| **NacS** | 0.90 | ± | 0.02 | **1.13^ΦΦΦ^** | ± | 0.03 | **1.00^Δ^** | ± | 0.03 | **1.05^Δ^** | ± | 0.02 | 1 | 14 | 11 | -7 | 25 | **5^###^** |
| *Basal Ganglia* | | | | | | | | | | | | | | | | | | |
| **VMST** | 1.27 | ± | 0.04 | 1.34 | ± | 0.02 | 1.31 | ± | 0.04 | 1.38 | ± | 0.02 | 3 | **6*** | 3 | 3 | 6 | 5 |
| **DLST** | 1.31 | ± | 0.02 | 1.32 | ± | 0.02 | 1.27 | ± | 0.03 | 1.38 | ± | 0.02 | 1 | **5*** | -2 | 5 | 1 | 8 |
| **GP** | 0.83 | ± | 0.03 | 0.79 | ± | 0.03 | 0.83 | ± | 0.02 | 0.74 | ± | 0.01 | -3 | **-8**** | 0 | -6 | -5 | -11 |
| **SNR** | 0.81 | ± | 0.01 | 0.77 | ± | 0.02 | 0.76 | ± | 0.03 | 0.75 | ± | 0.02 | -5 | -4 | -7 | -3 | -6 | -2 |
| **SNC** | 0.97 | ± | 0.03 | 0.84 | ± | 0.02 | 0.95 | ± | 0.02 | 0.81 | ± | 0.02 | -3 | **-14***** | -2 | -4 | -14 | -15 |
| *Amygdala* | | | | | | | | | | | | | | | | | | |
| **BLA** | 0.71 | ± | 0.03 | 0.78 | ± | 0.03 | 0.78 | ± | 0.03 | 0.80 | ± | 0.02 | 6 | 6 | 9 | 3 | 9 | 3 |
| **meA** | 0.65 | ± | 0.03 | 0.74 | ± | 0.02 | 0.72 | ± | 0.03 | 0.68 | ± | 0.03 | 0 | 4 | 11 | -9 | 14 | **-6^#^** |
| **CeA** | 0.73 | ± | 0.02 | **0.84^Φ^** | ± | 0.03 | **0.84^ΔΔ^** | ± | 0.02 | 0.83 | ± | 0.02 | **7*^‡^*** | 6 | 15 | -1 | 14 | **-1^#^** |

*Data shown as the Mean ± S.E.M. ^14^C-2-deoxyglucose uptake ratio and relevant % changes. *denotes p<0.05, **denotes p<0.01 and ***denotes p<0.001 significant treatment effect (2-way ANOVA, no genotype x treatment interaction). ^+^denotes p<0.05 and ^++^denotes p<0.01 significant genotype effect (2-way ANOVA, no genotype x treatment interaction). ^#^denotes p<0.05, ^##^denotes p<0.01 and ^###^denotes p<0.001 significant genotype x treatment interaction (2-way ANOVA).^Φ^denotes p<0.05, ^ΦΦ^denotes p<0.01 and ^ΦΦΦ^denotes p<0.001 significant difference from saline control (within genotype, t-test with post-hoc Bonferroni-Holm correction). ^Δ^denotes p<0.05, ^ΔΔ^denotes p<0.01 and ^ΔΔΔ^denotes p<0.001 significant genotype effect (within treatment group, t-test with Bonferroni-Holm correction). Post-hoc results are shown only for regions in which a significant genotype x treatment interaction was found by 2-way ANOVA.*

Table S2(E). Overt alterations in local cerebral glucose utilization in saline and ketamine treated *Disc1tr* Hemi mice: auditory, septum/DB and neuromodulatory regions

| **Region** | **Wild-type (Wt)** | | | | | | ***Disc1tr* Hemi** | | | | | | **% Genotype Effect (Treatments pooled)** | **% Ketamine Effect (Genotypes pooled)** | **% Genotype Effect** | | **% Ketamine Effect** | |
| --- | --- | --- | --- | --- | --- | --- | --- | --- | --- | --- | --- | --- | --- | --- | --- | --- | --- | --- |
|  | **Saline** | | | **Ketamine** | | | **Saline** | | | **Ketamine** | | |  |  |  |  |  |  |
|  | **Mean** |  | **SEM** | **Mean** |  | **SEM** | **Mean** |  | **SEM** | **Mean** |  | **SEM** |  |  | **Saline** | **Ketamine** | **Wt** | ***Disc1tr* Hemi** |
| *Auditory* | | | | | | | | | | | | | | | | | | |
| **IC** | 2.06 | ± | 0.04 | 1.52 | ± | 0.05 | 1.98 | ± | 0.11 | 1.46 | ± | 0.05 | -4 | **-26***** | -4 | -4 | -26 | -26 |
| **MG** | 1.27 | ± | 0.03 | 0.96 | ± | 0.03 | 1.24 | ± | 0.04 | 1.01 | ± | 0.02 | 1 | **-21***** | -3 | 5 | -24 | -19 |
| **AudC** | 1.29 | ± | 0.02 | 1.22 | ± | 0.03 | 1.25 | ± | 0.05 | 1.21 | ± | 0.01 | -2 | -4 | -4 | 0 | -6 | -3 |
| *Septum/ Diagonal Band of Broca (DB)* | | | | | | | | | | | | | | | | | | |
| **MS** | 1.00 | ± | 0.03 | 0.95 | ± | 0.02 | 1.05 | ± | 0.02 | 0.94 | ± | 0.01 | 2 | **-8**** | 5 | -2 | -5 | -10 |
| **LS** | 0.88 | ± | 0.03 | 0.84 | ± | 0.03 | 0.97 | ± | 0.03 | 0.90 | ± | 0.02 | **8^+^** | **-6*** | 10 | 7 | -5 | -7 |
| **VDB** | 0.99 | ± | 0.03 | 0.89 | ± | 0.03 | 1.03 | ± | 0.01 | 0.86 | ± | 0.03 | 1 | **-14***** | 4 | -3 | -11 | -16 |
| **HDB** | 1.16 | ± | 0.03 | 1.01 | ± | 0.03 | 1.14 | ± | 0.02 | 1.01 | ± | 0.01 | -1 | **-12***** | -1 | 0 | -12 | -12 |
| *Neuromodulatory* | | | | | | | | | | | | | | | | | | |
| **DR** | 0.83 | ± | 0.03 | 0.74 | ± | 0.02 | 0.79 | ± | 0.03 | 0.72 | ± | 0.04 | -4 | **-10*** | -5 | -3 | -11 | -9 |
| **MR** | 1.08 | ± | 0.04 | 1.01 | ± | 0.03 | 1.07 | ± | 0.04 | 0.95 | ± | 0.04 | -3 | **-9**** | -1 | -6 | -6 | -11 |
| **VTg** | 1.25 | ± | 0.03 | 1.06 | ± | 0.02 | 1.16 | ± | 0.04 | 0.98 | ± | 0.04 | **-7^+^** | **-15***** | -7 | -7 | -15 | -15 |
| **DTg** | 1.33 | ± | 0.04 | 1.22 | ± | 0.03 | 1.26 | ± | 0.03 | 1.07 | ± | 0.04 | **-8^++^** | **-12***** | -6 | -12 | -9 | -15 |
| **LC** | 1.06 | ± | 0.01 | 0.97 | ± | 0.04 | 0.91 | ± | 0.03 | 0.85 | ± | 0.03 | **-13^+++^** | **-7*** | -13 | -12 | -8 | -7 |

*Data shown as the Mean ± S.E.M. ^14^C-2-deoxyglucose uptake ratio and relevant % changes. *denotes p<0.05, **denotes p<0.01 and ***denotes p<0.001 significant treatment effect (2-way ANOVA, no genotype x treatment interaction). ^+^denotes p<0.05 and ^++^denotes p<0.01 significant genotype effect (2-way ANOVA, no genotype x treatment interaction). ^#^denotes p<0.05 significant genotype x treatment interaction (2-way ANOVA).^Φ^denotes p<0.05, ^ΦΦ^denotes p<0.01 and ^ΦΦΦ^denotes p<0.001 significant difference from saline control (within genotype, t-test with post-hoc Bonferroni-Holm correction). ^Δ^denotes p<0.05, ^ΔΔ^denotes p<0.01 and ^ΔΔΔ^denotes p<0.001 significant genotype effect (within treatment group, t-test with Bonferroni-Holm correction). Post-hoc results are shown only for regions in which a significant genotype x treatment interaction was found by 2-way ANOVA.*

Table S2(F). Overt alterations in local cerebral glucose utilization in saline and ketamine treated *Disc1tr* Hemi mice: hippocampal and multimodal regions

| **Region** | **Wild-type** | | | | | | **Hemi** | | | | | | **% Genotype Effect (Treatments pooled)** | **% Ketamine Effect (Genotypes pooled)** | **% Genotype *Disc1tr* Effect** | | **% Ketamine Effect** | |
| --- | --- | --- | --- | --- | --- | --- | --- | --- | --- | --- | --- | --- | --- | --- | --- | --- | --- | --- |
|  | **Saline** | | | **Ketamine** | | | **Saline** | | | **Ketamine** | | |  |  |  |  |  |  |
|  | **Mean** |  | **SEM** | **Mean** |  | **SEM** | **Mean** |  | **SEM** | **Mean** |  | **SEM** |  |  | **Saline** | **Ketamine** | **Wt** | ***Disc1tr* Hemi** |
| *Hippocampus* | | | | | | | | | | | | | | | | | | |
| **DSub** | 1.22 | ± | 0.03 | 1.39 | ± | 0.07 | 1.14 | ± | 0.03 | 1.20 | ± | 0.03 | **-10^++^*^‡^*** | **10*** | -7 | -13 | 14 | 6 |
| **CA1** | 0.98 | ± | 0.05 | 0.90 | ± | 0.06 | 0.92 | ± | 0.04 | 0.82 | ± | 0.03 | -7 | **-10*** | -6 | -8 | -9 | -11 |
| **CA2** | 0.84 | ± | 0.03 | 0.77 | ± | 0.03 | 0.82 | ± | 0.04 | 0.74 | ± | 0.02 | -3 | **-9*** | -2 | -4 | -8 | -10 |
| **CA3** | 0.66 | ± | 0.02 | 0.63 | ± | 0.02 | 0.72 | ± | 0.04 | 0.62 | ± | 0.01 | 4 | **-9**** | 9 | -1 | -5 | -13 |
| **DG** | 0.64 | ± | 0.01 | **0.72^Φ^** | **±** | **0.02** | 0.65 | ± | 0.03 | **0.65^Δ^** | **±** | **0.01** | -5 | 6 | 1 | -10 | 12 | **-1^#^** |
| **ML** | 1.09 | ± | 0.03 | 1.15 | ± | 0.03 | 1.04 | ± | 0.03 | 1.12 | ± | 0.04 | -3 | **6*** | -4 | -2 | 5 | 8 |
| *Multimodal* | | | | | | | | | | | | | | | | | | |
| **Hab** | 1.13 | ± | 0.06 | 0.92 | ± | 0.04 | 0.96 | ± | 0.02 | 0.85 | ± | 0.03 | **-12^++^** | **-15***** | -15 | -8 | -19 | -12 |
| **MB** | 1.80 | ± | 0.06 | 2.23 | ± | 0.11 | 1.76 | ± | 0.09 | 2.14 | ± | 0.10 | -3 | **23***** | -2 | -4 | 24 | 22 |
| **CC** | 0.43 | ± | 0.03 | 0.48 | ± | 0.03 | 0.40 | ± | 0.07 | 0.41 | ± | 0.02 | -11 | 8 | -6 | -15 | 14 | 3 |

*Data shown as the Mean ± SEM ^14^C-2-deoxyglucose uptake ratio and relevant % changes. *denotes p<0.05, **denotes p<0.01 and ***denotes p<0.001 significant treatment effect (2-way ANOVA, no genotype x treatment interaction). ^+^denotes p<0.05 and ^++^denotes p<0.01 significant genotype effect (2-way ANOVA, no genotype x treatment interaction). ^#^denotes p<0.05 significant genotype x treatment interaction (2-way ANOVA).^Φ^denotes p<0.05, ^ΦΦ^denotes p<0.01 and ^ΦΦΦ^denotes p<0.001 significant difference from saline control (within genotype, t-test with post-hoc Bonferroni-Holm correction). ^Δ^denotes p<0.05, ^ΔΔ^denotes p<0.01 and ^ΔΔΔ^denotes p<0.001 significant genotype effect (within treatment group, t-test with Bonferroni-Holm correction). Post-hoc results are shown only for regions in which a significant genotype x treatment interaction was found by 2-way ANOVA.*

Table S3. Hub region identification and alterations in regional importance identified through centrality analysis

*Data shown as the z-score for each centrality measure in real brain networks relative to 11,000 calibrated Erdös-Rényi networks. Bold denotes the centrality measure in which a brain region is considered to be an important hub in the brain network. *denotes p<0.05 significant difference in regional centrality between Disc1tr Hemi and wild-type mice, determined by comparison of the real z-score difference to that in 11,000 random permutations of the real data. The identities of each abbreviated brain region are shown in Table S2(A).*

Table S4. Altered functional connectivity of the anterior Prelimbic Cortex (aPrL) in *Disc1tr* Hemi mice

*Data shown as the Mean ± S.E.M. of the variable importance to the projection (VIP) statistic for each region generated using the PLSR algorithm as previously outlined (Dawson et al., 2012). Bold values denote VIP’s considered to be functionally connected to the seed region (95% confidence interval of VIP statistic exceeds the 0.8 threshold). *denotes p<0.05, **denotes p<0.01 significant increase in connectivity as compared to wild-type animals (t-test with Bonferroni correction). The identities of each abbreviated brain region are shown in Table S2(A).*

Table S5. Altered functional connectivity of the lateral orbital cortex (LO) in *Disc1tr* Hemi mice

*Data shown as the mean ± S.E.M. of the variable importance to the projection (VIP) statistic for each region generated using the PLSR algorithm as previously outlined (Dawson et al., 2012). Bold values denote VIP’s considered to be functionally connected to the seed region (95% confidence interval of VIP statistic exceeds the 0.8 threshold). *denotes p<0.05, **denotes p<0.01 significant increase in connectivity as compared to wild-type animals (t-test with Bonferroni correction). The identities of each abbreviated brain region are shown in Table S2(A).*

Table S6. Altered functional connectivity of the ventral orbital cortex (VO) in *Disc1tr* Hemi mice

*Data shown as the mean ± S.E.M. of the variable importance to the projection (VIP) statistic for each region generated using the PLSR algorithm as previously outlined (Dawson et al., 2012). Bold values denote VIP’s considered to be functionally connected to the seed region (95% confidence interval of VIP statistic exceeds the 0.8 threshold). *denotes p<0.05, **denotes p<0.01 significant increase in connectivity as compared to wild-type animals (t-test with Bonferroni correction). The identities of each abbreviated brain region are shown in Table S2(A).*

Table S7. Altered functional connectivity of the anteromedial thalamus (AMthal) in *Disc1tr* Hemi mice

*Data shown as the mean ± S.E.M. of the variable importance to the projection (VIP) statistic for each region generated using the PLSR algorithm as previously outlined (Dawson et al., 2012). Bold values denote VIP’s considered to be functionally connected to the seed region (95% confidence interval of VIP statistic exceeds the 0.8 threshold). *denotes p<0.05, **denotes p<0.01 significant increase in connectivity as compared to wild-type animals (t-test with Bonferroni correction). The identities of each abbreviated brain region are shown in Table S2(A).*

Table S8. Altered functional connectivity of the dorsal reticular thalamus (dRT) in *Disc1tr* Hemi mice

*Data shown as the mean ± S.E.M. of the variable importance to the projection (VIP) statistic for each region generated using the PLSR algorithm as previously outlined (Dawson et al., 2012). Bold values denote VIP’s considered to be functionally connected to the seed region (95% confidence interval of VIP statistic exceeds the 0.8 threshold). *denotes p<0.05, **denotes p<0.01 significant increase in connectivity as compared to wild-type animals (t-test with Bonferroni correction). The identities of each abbreviated brain region are shown in Table S2(A).*

Table S9. Altered functional connectivity of the hippocampal CA1 (CA1) in *Disc1tr* Hemi mice

*Data shown as the mean ± S.E.M. of the variable importance to the projection (VIP) statistic for each region generated using the PLSR algorithm as previously outlined (Dawson et al., 2012). Bold values denote VIP’s considered to be functionally connected to the seed region (95% confidence interval of VIP statistic exceeds the 0.8 threshold). *denotes p<0.05, **denotes p<0.01 significant increase in connectivity as compared to wild-type animals (t-test with Bonferroni correction). The identities of each abbreviated brain region are shown in Table S2(A).*

Table S10. Altered functional connectivity of the horizontal DB (HDB) in *Disc1tr* Hemi mice

*Data shown as the mean ± S.E.M. of the variable importance to the projection (VIP) statistic for each region generated using the PLSR algorithm as previously outlined (Dawson et al., 2012). Bold values denote VIP’s considered to be functionally connected to the seed region (95% confidence interval of VIP statistic exceeds the 0.8 threshold). *denotes p<0.05, **denotes p<0.01 significant increase in connectivity as compared to wild-type animals (t-test with Bonferroni correction). The identities of each abbreviated brain region are shown in Table S2(A).*

Table S11. Altered functional connectivity of the corpus callosum (CC) in *Disc1tr* Hemi mice

*Data shown as the mean ± S.E.M. of the variable importance to the projection (VIP) statistic for each region generated using the PLSR algorithm as previously outlined (Dawson et al., 2012). Bold values denote VIP’s considered to be functionally connected to the seed region (95% confidence interval of VIP statistic exceeds the 0.8 threshold). *denotes p<0.05, **denotes p<0.01 significant increase in connectivity as compared to wild-type animals (t-test with Bonferroni correction). The identities of each abbreviated brain region are shown in Table S2(A).*

Table S12. Altered functional connectivity of the habenula (Hab) in *Disc1tr* Hemi mice

*Data shown as the mean ± S.E.M. of the variable importance to the projection (VIP) statistic for each region generated using the PLSR algorithm as previously outlined (Dawson et al., 2012). Bold values denote VIP’s considered to be functionally connected to the seed region (95% confidence interval of VIP statistic exceeds the 0.8 threshold). *denotes p<0.05, **denotes p<0.01 significant increase in connectivity as compared to wild-type animals (t-test with Bonferroni correction). The identities of each abbreviated brain region are shown in Table S2(A).*

Table S13. Passive membrane and action potential waveform properties of medial prefrontal cortex pyramidal neurones in *Disc1*tr Hemi mice

|  | **Wild-type** | | ***Disc1tr* Hemi** | |
| --- | --- | --- | --- | --- |
|  | RS (n=24) | IB (n=7) | RS (n=28) | IB (n=4) |
| RMP (mV) | -88.5 ± 0.6 | -87.1 ± 0.9 | -88.2 ± 0.5 | -88.2 ± 0.5 |
| R_I_ (MΩ) | 379 ± 20 | 379 ± 40 | 344 ± 21 | 259 ± 29 |
| τ_M_ (ms) | 18.4 ± 0.8 | 18.5 ± 1.2 | 16.9 ± 0.7 | 16.7 ± 0.5 |
| % sag | 16.3 ± 1.2 | 14.4 ± 2.6 | 14.2 ± 1.0 | 8.3 ± 1.9 |
| Action potential |  |  |  |  |
| Peak (mV) | 36 ± 1 | 31 ± 3 | 35 ± 1 | 34 ± 2 |
| Max dV/dt (V/s) | 466 ± 18 | 374 ± 20 | 443 ± 13 | 397 ± 40 |
| Width (ms) | 0.83 ± 0.01 | 0.84 ± 0.04 | 0.80 ± 0.01 | 0.89 ± 0.08 |
| Threshold (mV) | -55.5 ± 0.4 | -51.0 ± 0.9 | -54.9 ± 0.6 | -53.9 ± 1.1 |

*Data shown as mean ± S.E.M. medial prefrontal cortex (mPFC) pyramidal neurons were classified as either regular spiking (RS) or intrinsic bursting (IB), depending on the respective absence or presence of a fast afterdepolarising potential. RMP, resting membrane potential; R_I_, input resistance; τ_M_, membrane time constant. All parameters (except RMP) were measured at a fixed membrane potential of -80 mV. No significant difference between the different classes of neurons or genotypes were observed in any parameter (P>0.1).*

Figure S1. Neuronal excitability properties in both regular spiking (RS) and intrinsic bursting (IB) neurons are genotype-independent in deep layer mPFC neurons


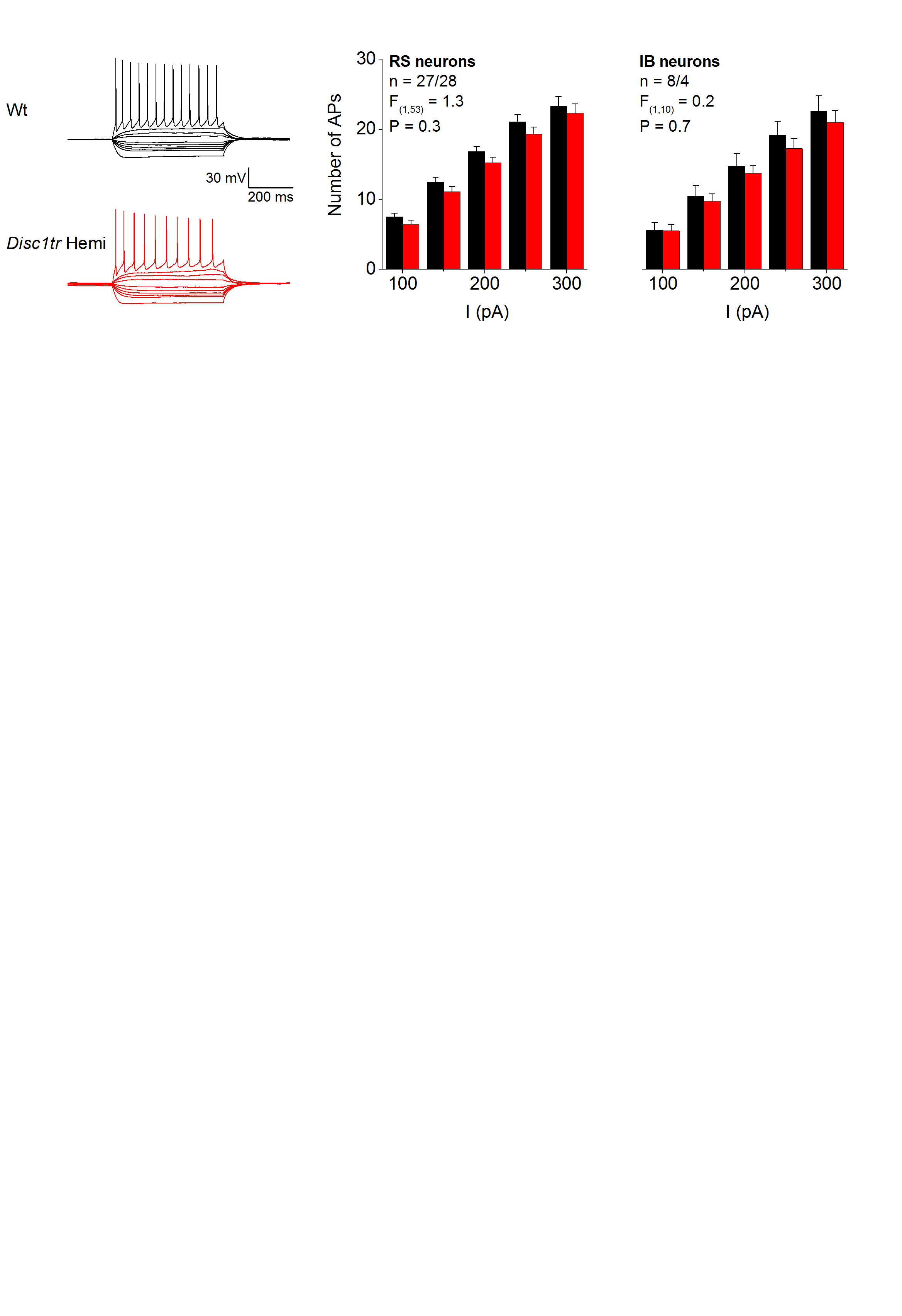


*Traces are representative current clamp recordings of layer V/VI medial prefrontal cortex (mPFC) neurons showing the voltage deflections in response to hyperpolarising and depolarising current injections (between 100 and 300 pA). The hisograms depict the mean number of action potentials (APs) fired in response to depolarising current injections in wild-type (Wt, black) and Disc1tr* *Hemi (red) deep layer regular spiking (RS; left) and intrinsic bursting (IB; right) neurons.*

Figure S2. Miniature synaptic currents in deep layer medial prefrontal cortex (mPFC) neurons are unaffected in *Disc1tr* Hemi mice


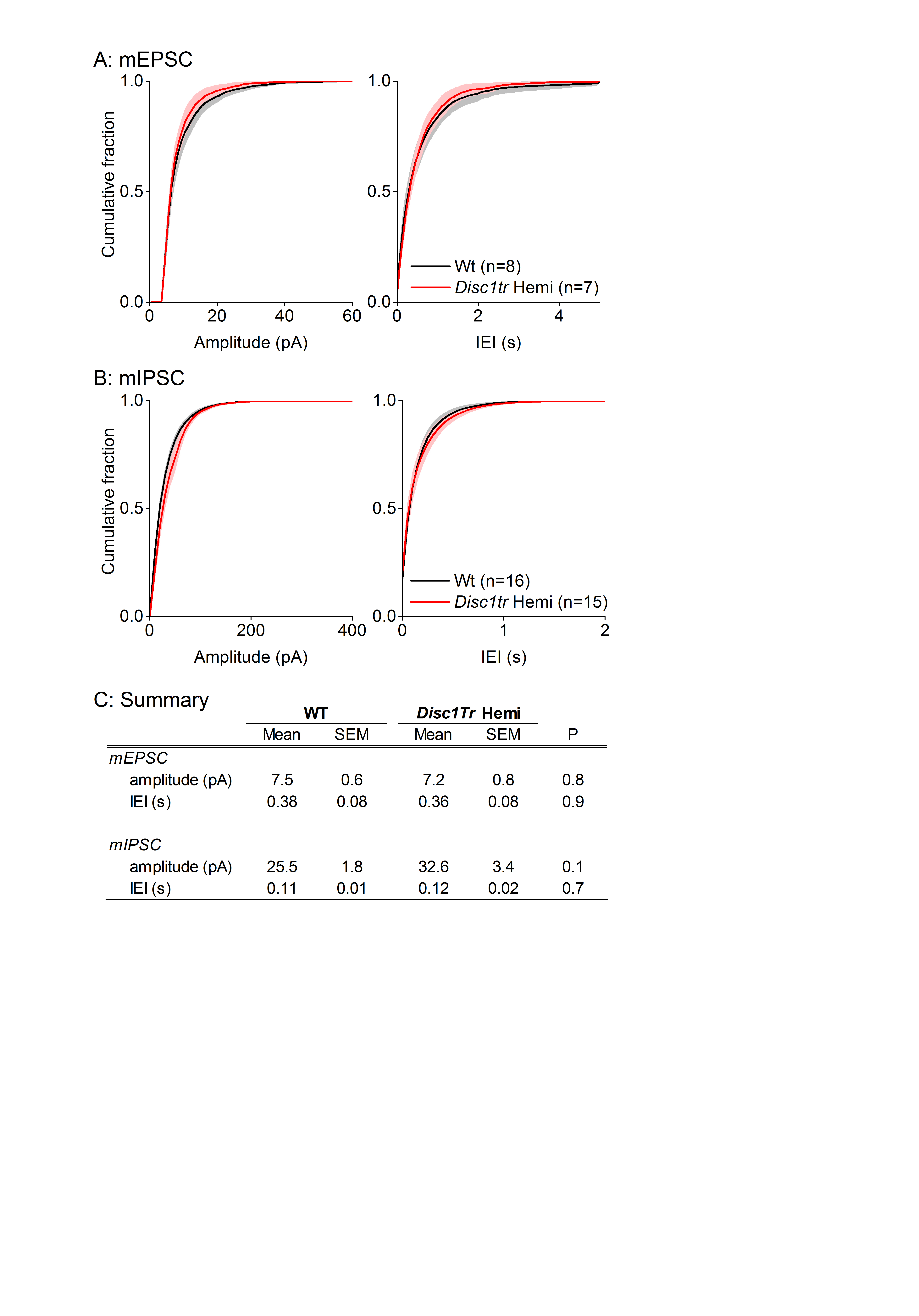


*Cumulative probability distributions of miniature excitatory and inhibitory post synaptic current (mEPSC and mIPSC, respectively) amplitude (left) and inter-event interval (IEI; right). All mEPSCs and mIPSCs were recorded in the presence of 500 nM TTX. The thick line is the mean distribution, whilst the shaded area represents the S.E.M. C: Table summarising the mean of the median amplitudes and IEIs for mEPSCs and mIPSCs recorded from WT and and Disc1Tr Hemi neurons.*

**Dawson *et al*., 2015. Supplemental Methods**

**^14^C-2-DG Autoradiographic Functional Brain Imaging**

Access to food was restricted 4-5 hours prior to ^14^C-2-DG imaging to obviate the potential influence of ketamine treatment on plasma glucose levels. One minute after either (R,S)-ketamine (30 mg.kg^-1^, *i.p*., 2 mls.kg^-1^) or saline treatment mice were injected with 4.625 MBq.kg^-1^ of ^14^C-2-DG (Perkin Elmer, UK) steadily over 10 seconds before being returned to the home cage. Forty five minutes after isotope injection animals were decapitated and a terminal blood sample collected by torso inversion. The brain was rapidly dissected out and frozen in isopentane (-40^o^C) and stored at -80^o^C until sectioning. Plasma glucose levels in the terminal blood sample were determined from a small sample (<10 µl) of the whole blood (Accu-Chek Aviva glucose monitor). For the determination of plasma ^14^C levels blood samples were centrifuged to separate the plasma and 20 µl aliquots were analyzed by liquid scintillation analysis (Packard), in triplicate. Frozen brains were sectioned (20 µm) in the coronal plane in a cryostat (-20^o^C). A series of three consecutive sections were retained from every 60 µm, thaw mounted onto slide covers and rapidly dried on a hot plate (70^o^C). Autoradiograms were generated by apposing these sections, together with precalibrated ^14^C-standards (40-1069 nCi/g tissue equivalents: Amersham International, UK) to X-ray film (Kodak, Biomax, MR) for 5 days. Autoradiographic images were analyzed by a computer-based image analysis system (MCID/M5+). The local isotope concentration for each brain region of interest (RoI) was derived from the optical density of autoradiographic images relative to that of the co-exposed ^14^C standards. Fifty eight anatomically distinct RoI were measured with reference to a stereotaxic mouse brain atlas (Paxinos and Franklin, 2001). Local cerebral glucose utilization (LCGU) in each RoI was determined as the ratio of ^14^C present in that region relative to the average ^14^C concentration in the whole brain of the same animal, referred to as the ^14^C-2-DG uptake ratio. Whole brain average ^14^C levels were determined from the average ^14^C concentration across all sections in which a RoI was measured.

In keeping with previous studies of similar experimental design the significance of changes in LCGU between experimental groups in anatomically discrete RoI were assumed to represent independent variables and no correction was made for multiple comparisons (see McCulloch *et al*., 1982 for discussion). Overt alterations in regional LCGU were analyzed using 2-way ANOVA with genotype (Wt, *Disc1tr* Hemi) and treatment (saline, ketamine) as independent variables after a first pass analysis confirmed that sex did not significantly alter constitutive LCGU, the impact of *Disc1* truncation on LCGU or the impact of acute ketamine treatment on LCGU. *Post-hoc* analysis, pairwise t-test with Bonferroni-Holm correction, was used to identify a significant genotype or treatment effect if a significant genotype *x* treatment interaction was identified in a given RoI.

**Analysis of Functional Brain Network Connectivity**

The inter-regional Pearson’s correlation coefficient was used as the metric of functional association between brain RoI, generated from the ^14^C-2-DG uptake ratios for each RoI across all animals within the same experimental group (i.e. *Disc1tr* Hemi mice or their Wt littermates). These correlations were Fisher z-transformed to give the correlation data a normal distribution. This resulted in a pair of 58 x 58 partial correlation matrices for each experimental group, each within group matrix representing the specific association strength between each of the 3,306 possible pairs of regions. From each correlation matrix (R) we derived a binary adjacency matrix (A) where the functional connection between two regions (a_i,j_ element) was zero if the Pearson’s correlation coefficient was lower than the defined threshold p_[i,j]_<T) and unity if the coefficient was greater than or equal to the defined threshold (p_[i,j]_≥T). The adjacency matrix can also be represented as an undirected graph *G*, where a line or edge represents the functional interaction between two brain RoI (nodes) if the partial correlation coefficient exceeds the defined threshold value.

Network science algorithms were applied to characterise the properties of the functional brain networks in *Disc1tr* Hemi mice and their Wt littermates at the global and regional scales. Data were analyzed using the igraph package (Càsrdi and Nepusz., 2006) in R (R Development Core Team, 2011). Global network architecture was quantified in terms of the mean degree (<k>), average path length (L_p_) and the mean cluster coefficient (C_p_) of the whole brain network. These measures quantitatively define global brain network connectivity, efficiency and clustering, respectively. Regional properties were defined in terms of degree (k), betweenness (B_c_) and eigenvector (E_c_) centrality. These measures allow us to quantitatively define which brain regions are hubs and which are exteriorities in each of the brain networks and how centrality status is significantly altered as a result of *Disc1* truncation. Global and regional metrics were determined on binary adjacency matrices generated over a range of correlation thresholds (Pearson’s r=0.4 to 0.5 at 0.01 intervals, Fisher’s z=0.4236 to 0.5493) that generated fully connected networks for each group. Regional metrics were determined by comparing regional centrality measures in real networks to those in calibrated (Erdös-Rényi) random graphs (1,000 random graphs at each correlation threshold, 11,000 random graphs in total).

Here we provide brief, formal definitions of the global network metrics determined in this study, including mean degree (<k>), average path length (L_p_) and mean clustering co-efficient (C_p_). The degree of a node (k) is simply the number of edges that connect that node to the network, so highly connected nodes have a high degree. The mean degree (<K>, equation [1]) is the average number of edges of all the nodes in the network. A sparse network therefore has a low mean degree.

(1)

The minimum path length between two nodes in a graph (L_i,j_) is the smallest number of edges that must be traversed to make a connection between them. If two nodes are immediate neighbours, directly connected by a single edge, then L_i,j_=1. The average path length (L_p_, equation [2]), or average L_i,j_ across all possible pairs, is the average number of steps along the shortest paths for all nodes in the network. This provides a measure of global network efficiency, where networks with a low average path length allow more efficient information transfer across the network.

(2)

The clustering coefficient (C) for a given node is the fraction of pairs of neighbours that are themselves connected. It provides an indication of how well-connected the neighbourhood of a node is. The mean clustering coefficient (C_p_, equation [3]) is the average clustering coefficient of all of the nodes in the network, which provides a measure of the local density or cliquishness of the network. A high mean clustering coefficient suggests high clustering and therefore efficient local information transfer.

(3)

The significance of *Disc1* mutation-induced alterations in the global properties of 2-DG functional brain networks was determined by comparison of the real difference in each measure to that of 5,000 random permutations of the data at each correlation threshold (55,000 random permutations in total). Significance was set at *p*<0.05 and was determined from the average *p*-value determined across the entire correlation threshold range analyzed.

**Regional Centrality and Hub Brain Region Identification**

In this study we consider node centrality as determined by degree (k_i_), betweenness (B_c_) and eigenvector centrality (E_c_). Degree centrality is based upon the number of nodal connections. Betweenness centrality (B_c_, equation [4]) is based the fraction of shortest paths that go through a given node, *i*.

(4)

Here σ_st_ denotes the total number of shortest paths from *s* to *t* and σ_st_(*i*) denotes the number of shortest paths from *s* to *t* that go through a node (*i*). Eigenvector centrality (E_c_, equation [5]) is based upon the number of connections a given nodes connected neighbours have.


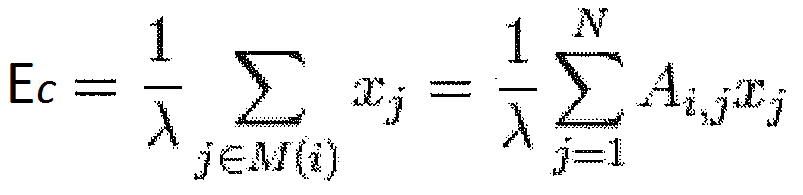
 (5)

A brain region may be considered to be an important hub region in the network if it has a high degree, betweenness or eigenvector centrality. In this study a brain region was defined as a hub, in the 2-DG functional brain network of either *Disc1* mutant or Wt animals, if, for any centrality measure, the regional centrality measure in the real network relative to that of calibrated random Erdös-Rényi graphs (1,000 random graphs at each correlation threshold, 11,000 random graphs in total), was z>1.96. The z-score for each centrality measure was calculated as shown in equation 6. The formula below outlines the z-score calculation for betweenness centrality (B_c_) of node *i* for illustrative purposes. Alternatively, a brain region may be considered to be an exteriority, with little influence in the network, if its degree, betweenness or eigenvector centrality is significantly lower than that seen for nodes in calibrated random graphs (z < -1.96).

(6)

The significance of *Disc1* mutation-induced alterations in regional centrality was determined by comparison of the z-score difference in regional centrality in the real brain networks relative to that in 11,000 random permutations of the data (1,000 random permutations at each correlation threshold). Significance was set at *p*<0.05.

**Determination of Regional Functional Connectivity**

Following the identification of *Disc1tr Hemi*-induced alterations in regional importance (centrality), we sought to characterise the alterations in regional functional connectivity that underlie these alterations. To achieve this we employed the PLSR algorithm to define significant differences in the functional connectivity of defined “seed” brain RoIs to all other RoI analyzed. The application of PLSR to functional 2-DG brain imaging data and its interpretation were previously outlined (see Dawson *et al*., 2012; 2013) and the algorithm itself was discussed in detail by Wold *et al*., 2001. Data were analyzed using the PLS package (Mevik *et al*., 2011) in R (R Development Core Team, 2011). Here, the selection of “seed” brain RoIs was guided by the observed significant alterations in regional centrality, as defined through network analysis. For regions showing a significant increase in regional importance in the *Disc1* mutant only significant increase in regional functional connectivity were considered. Likewise, for regions showing a significant decrease in regional importance in the *Disc1* mutant only a significant decrease in regional functional connectivity was considered. As previously outlined (Dawson *et al*., 2012; 2013) “seed” regional functional connectivity (dependent variable in the PLSR model) to all other RoI analyzed (explanatory variables; 57) was considered in terms of the variable importance to the projection (VIP) statistic. Within each experimental group, and for each “seed” region, a significant functional connection was considered to exist if the 95% confidence interval of the VIP statistic exceeded 0.8, as this threshold denotes a considerable contribution of the explanatory variable to the dependent variable in PLSR models (Wold *et al*., 2001). The standard deviation and confidence interval for the VIP statistic were estimated by jack-knifing. The significance of *Disc1* mutation induced alterations in region functional connectivity (the VIP statistic) were analyzed using t-tests with Bonferroni correction for multiple comparisons. Significance was set at *p*<0.05.

**Electrophysiology**

All electrophysiological recordings were made from male mice euthanized by cervical dislocation. Following subsequent decapitation the brain was rapidly removed and placed in ice-cold (~4 °C) sucrose-based solution comprising (in mM): sucrose, 189; D-glucose, 10; NaHCO_3_, 26; KCl, 3; MgSO_4_, 5; CaCl_2_, 0.1; NaH_2_PO_4_, 1.25. The caudal portion of the brain was removed with a coronal razor blade cut perpendicular to the rostro-caudal axis. The frontal portion of the brain was then mounted on a stainless steel plate which was held magnetically at an angle of ~10° from horizontal. Thus when sections were cut using a vibrotome, they were at an angle of ~10° from a true coronal section. Cutting slices at this angle maintains the axon tract connecting the hippocampus to the PFC (Parent *et al.,* 2010). Slices (300 μm thick) were cut using a Leica VT1200 vibratome and then transferred to a chamber filled with artificial cerebrospinal fluid (aCSF) comprising (mM): NaCl, 124; KCl, 3; NaHCO_3_, 26; CaCl_2_, 2; NaH_2_PO_4_, 1.25; MgSO_4_, 1; d-glucose, 10 and equilibrated with 95% O_2_ and 5% CO_2_. The slices were first warmed to 32 ± 1°C, over a period of 30 minutes and were subsequently stored at room temperature for at least another 30 minutes, prior to recording. Slices were then transferred, one at a time to a submerged recording chamber maintained at 32 ± 1°C, perfused (~2-3 ml.min^-1^) with aCSF.

Individual neurons in layer V or VI of the medial prefrontal cortex (mPFC, prelimbic or infralimbic cortex) were visualised using infra-red differential interference contrast optics. Whole cell current clamp recordings were made using borosilicate glass microelectrodes (3-5 MΩ) containing (mM): K-gluconate, 145; NaCl, 5; Na-HEPES, 10; EGTA, 0.2; Na-GTP, 0.3; Mg-ATP, 4. The pairing of our aCSF and electrode solution produced a liquid junction potential of 15 mV which was corrected for arithmetically. For mEPSC recordings, a Cesium MethylSulfonate (CsMeSO4) based internal solution was used, containing (mM): 130 CsMeSO4, 20 NaCl, 10 HEPES free acid, 0.2 EGTA, 0.3 GTP-Na salt, 4 ATP-Mg salt. For mIPSC recordings, a Cesium Chloride (CsCl) based electrode solution was used, containing (mM): 130 CsCl, 5 NaCl, 10 HEPES free acid, 0.2 EGTA, 5 QX314-Cl, 0.3 GTP-Na salt and 4 ATP-Mg salt. All solutions were adjusted to a pH 7.3 and had an osmolarity of 285-290 mOsm. Only cells with a junction potential-corrected resting membrane potential more negative than -60 mV were used for experiments.

Excitatory postsynaptic potentials (EPSPs) were elicited by stimulating the axon bundle ventral to the corpus callosum, using a concentric bipolar stimulating electrode (CBARB100, FHC) connected to an isolated stimulator. EPSPs recorded in response to this axon bundle are thought to be largely hippocampal in origin (Parent *et al*., 2010).

Current clamp recordings were made using the bridge circuit of a MultiClamp 700B amplifier (Molecular Devices, Union City, CA). Data were filtered at 10 kHz and digitized at 100 kHz and recorded to a personal computer using Clampex 10 software (Molecular Devices). Data were analyzed off-line using custom written scripts in Matlab.

**Determination of NMDA Receptor Subunit Expression**

Expression levels of NMDAR subunits GluN1, GluN2A, GluN2B and GluN3B were measured using standard western blot techniques. Briefly, adult *Disc1tr* Hemi (n=7) and wild-type (n=7) mice (re-derived from cryopreserved embryos at Jackson Labs, Bar Harbor, ME) were euthanized and the prefrontal cortex and hippocampus dissected out, frozen on dry ice, and kept at -80°C until use. Brain tissues were homogenized in lysis buffer (0.1% SDS and 50mM NaFl) containing protease and phosphatase inhibitors (Pierce Biotechnology, Rockford, IL, USA) and then centrifuged at 15,000*g* for 30 min. Samples (80 μg protein each) were separated using SDS–polyacrylamide gel electrophoresis (4-12% gradient gel NuPage Bis-Tris) and subsequently transferred to nitrocellulose membranes (0.22 μm; Invitrogen, CA, USA). The samples were then incubated overnight with the following antisera; anti-GluN1 (1:500; Santa Cruz sc-1467 goat polyclonal IgG and 1:1000; Millipore 05-432 mouse monoclonal), anti-GluN2A (1:500; Santa Cruz sc-1468 goat polyclonal IgG), anti-GluN2B (1:500; Santa Cruz sc-1469 goat polyclonal IgG and 1:1000; Millipore 5778 mouse monoclonal) and anti-GluN3B (1:1000; Abcam ab35677 rabbit polyclonal). The integrated intensity (I.I.) of specific protein bands were normalized to anti-GAPDH antibodies(1:10,000; Cell Signaling Technology #5174 rabbit polyclonal and 1:10,000; Sigma G8795 mouse monoclonal) at 4°C.   Afterward, the membranes were incubated with Alexa Fluor 700 and 800 conjugated with respective goat anti-rabbit, anti-mouse, or anti-goat antibody (1:10000; Invitrogen, Eugene, OR, USA) for 60 min. Detection and quantification of specific bands were performed using a fluorescence scanner (Odyssey Infrared Imaging System, LI-COR Biotechnology, Lincoln, NE, USA).

**Supplemental References**

Càsardi G, Nepusz T. The igraph package for complex network research. InterJournal Complex Systems, 1695. 2006. (http://igraph.org)

Dawson N, Thompson RJ, McVie A, Thomson DM, Morris BJ, Pratt JA. Modafinil reverses phencyclidine-induced deficits in cognitive flexibility, cerebral metabolism and functional brain connectivity. *Schizophr Bull* 2012; **38,** 457-474.

Dawson N, Morris BJ, Pratt JA. Subanaesthetic ketamine treatment alters prefrontal cortex connectivity with thalamus and ascending subcortical systems. *Schizophr Bull* 2013; **39:** 366-77.

McCulloch J, Kelly PAT, Ford I. Effect of apomorphine on the relationship between cerebral glucose utilization and local cerebral blood-flow (with an appendix on its statistical analysis). *J Cereb Blood Flow Metab* 1982; **2:** 487-499.

Mevik BH, Wehrens R, Liland KH. Pls: Partial Least Squares and Principle Component regression. R package version 2.3-0. 2011. <http://CRAN.R-project.org/package=pls>.

Parent MA, Wang L, Su J, Netoff T, Yuan LL. Identification of the hippocampal input to medial prefrontal cortex in vitro. *Cereb Cortex* 2010; **20:** 393-403.

Paxinos G, Franklin KBJ. The mouse brain in stereotaxic coordinates. Second Edition. 2001. Academic Press, UK.

R: A language and environment for statistical computing. R Foundation for statistical computing. R foundation for Statistical Computing, Vienna, Austria. ISBN 3-900051-07-0. <http://www.R-project.org/>.

Wold S, Sjostrom M, Eriksson L. PLS-regression: a basic tool of chemometrics. *Chemom Intell Lab Syst* 2001; **58:** 109-130.
